# A new GWAS and meta-analysis with 1000Genomes imputation identifies novel risk variants for colorectal cancer

**DOI:** 10.1038/srep10442

**Published:** 2015-05-20

**Authors:** Nada A. Al-Tassan, Nicola Whiffin, Fay J. Hosking, Claire Palles, Susan M. Farrington, Sara E. Dobbins, Rebecca Harris, Maggie Gorman, Albert Tenesa, Brian F. Meyer, Salma M. Wakil, Ben Kinnersley, Harry Campbell, Lynn Martin, Christopher G. Smith, Shelley Idziaszczyk, Ella Barclay, Timothy S. Maughan, Richard Kaplan, Rachel Kerr, David Kerr, Daniel D. Buchannan, Aung Ko Win, John Hopper, Mark Jenkins, Noralane M. Lindor, Polly A. Newcomb, Steve Gallinger, David Conti, Fred Schumacher, Graham Casey, Malcolm G. Dunlop, Ian P. Tomlinson, Jeremy P. Cheadle, Richard S. Houlston

**Affiliations:** 1 Department of Genetics, King Faisal Specialist Hospital and Research Center, P.O.Box 3354, Riyadh 11211, Saudi Arabia; 2 Division of Genetics and Epidemiology, The Institute of Cancer Research, London, UK; 3 Wellcome Trust Centre for Human Genetics and NIHR Comprehensive Biomedical Research Centre, Oxford, UK; 4 Colon Cancer Genetics Group, Institute of Genetics and Molecular Medicine, University of Edinburgh and MRC Human Genetics Unit, Western General Hospital Edinburgh, Crewe Road, Edinburgh, EH4 2XU, UK; 5 Institute of Cancer and Genetics, School of Medicine, Cardiff University, Heath Park, Cardiff, CF14 4XN, UK; 6 The Roslin Institute, University of Edinburgh, Easter Bush, Roslin, EH25 9RG, UK; 7 Centre for Population Health Sciences, University of Edinburgh, Edinburgh, EH8 9AG, UK; 8 CRUK/MRC Oxford Institute for Radiation Oncology, University of Oxford, Roosevelt Drive, Oxford, OX3 7DQ, UK; 9 MRC Clinical Trials Unit, Aviation House, 125 Kingsway, London, WC2B 6NH, UK; 10 Oxford Cancer Centre, Department of Oncology, University of Oxford, Churchill Hospital, Old Road, Headington, Oxford, OX3 7LE, UK; 11 Nuffield Department of Clinical Laboratory Sciences, University of Oxford, John Radcliffe Hospital, Headley Way, Oxford, OX3 9DU, UK; 12 Oncogenomics Group, Genetic Epidemiology Laboratory, Department of Pathology, The Univers‡ity of Melbourne, Victoria, Australia; 13 Centre for Epidemiology and Biostatistics, The University of Melbourne, Victoria, Australia; 14 Department of Health Sciences Research, Mayo Clinic, Scottsdale, AZ, USA; 15 Cancer Prevention Program, Fred Hutchinson Cancer Research Center, Seattle, WA, USA; 16 Samuel Lunenfeld Research Institute, Mount Sinai Hospital, Toronto, ON, Canada; 17 Department of Preventive Medicine, University of Southern California, Los Angeles, CA, USA

## Abstract

Genome-wide association studies (GWAS) of colorectal cancer (CRC) have identified 23 susceptibility loci thus far. Analyses of previously conducted GWAS indicate additional risk loci are yet to be discovered. To identify novel CRC susceptibility loci, we conducted a new GWAS and performed a meta-analysis with five published GWAS (totalling 7,577 cases and 9,979 controls of European ancestry), imputing genotypes utilising the 1000 Genomes Project. The combined analysis identified new, significant associations with CRC at 1p36.2 marked by rs72647484 (minor allele frequency [MAF] **=** 0.09) near *CDC42* and *WNT4* (*P* **=** 1.21 **×** 10^−8^, odds ratio [OR] **=** 1.21 ) and at 16q24.1 marked by rs16941835 (MAF **=** 0.21, *P* **=** 5.06 **×** 10^−8^; OR **=** 1.15) within the long non-coding RNA (lncRNA) RP11-58A18.1 and ~500 kb from the nearest coding gene *FOXL1*. Additionally we identified a promising association at 10p13 with rs10904849 intronic to *CUBN* (MAF = 0.32, *P* = 7.01 × 10^-8^; OR = 1.14). These findings provide further insights into the genetic and biological basis of inherited genetic susceptibility to CRC. Additionally, our analysis further demonstrates that imputation can be used to exploit GWAS data to identify novel disease-causing variants.

Twin studies indicate that heritable factors account for 35% of the variation in risk of developing colorectal cancer (CRC)^1^. However, only 5% of CRC can be attributed to the inheritance of high-penetrance mutations in the known genes^2^,^3^. Genome-wide association studies (GWAS) conducted primarily in European^4^,^5^,^6^,^7^,^8^,^9^,^10^,^11^,^12^ but also Asian^13^,^14^,^15^,^16^ populations have vindicated the long-held belief that part of the heritable risk of CRC is attributable to common, low-risk variants. These GWAS have provided insights into the biological basis of CRC, highlighting the role of genes within the bone morphogenetic protein signalling pathway (*BMP2, BMP4, GREM1* and *SMAD7*)^4^,^11^ and some candidate genes (*e.g*. *CDH1/CDH3*), as well as genes not previously implicated in CRC (*e.g*. *POLD3, TERC, CDKN1A* and *SHROOM2*)^5^,^6^.

Despite the success of GWAS the risk SNPs so far identified in European populations account for only 8% of the familial CRC risk (Supplementary Table 1). Together with the over-representation of association signals in GWAS strongly suggests that additional risk SNPs remain to be discovered. The statistical power of individual GWAS is limited by the modest effect sizes of the genetic variants and the requirement for a stringent threshold to establish statistical significance in order to avoid type 1 errors. Meta-analysis of GWAS data therefore offers the opportunity to identify new CRC risk loci and provide further insights into tumour biology. Furthermore, imputation of untyped variants in GWAS data using publicly available reference datasets increases the number of variants that can be tested for an association with CRC risk.

To identify new CRC susceptibility loci, we conducted an independent primary scan of CRC using patient samples from the COIN trial and performed a genome-wide meta-analysis with five previously published GWAS. To recover untyped genotypes, thereby maximising the prospects of identifying risk variants, we imputed over 10 million SNPs in the six GWAS datasets, using data from the 1000 Genomes Project^17^ as reference (see Materials & Methods for details).

## Methods

### Primary GWAS

The COIN GWAS was based on 2,244 CRC cases (64% male, mean age 61 years, SD = 10) ascertained through two independent Medical Research Council clinical trials of advanced/metastatic CRC; COIN and COIN-B^18^. Cases were genotyped using Affymetrix Axiom Arrays according to the manufacturer’s recommendations (Affymetrix, Santa Clara, CA 95051, USA), using duplicate samples and sequencing of significantly associated SNPs in a subset of samples to confirm genotyping accuracy. For all SNPs >99% concordant results were obtained. For controls, we made use of Wellcome Trust Case Control Consortium 2 (WTCCC2) Affymetrix 6.0 array data on 2,674 individuals from the UK Blood Service Control Group. Individuals were excluded with: <95% successfully genotyped SNPs (n = 122), discordant sex information (n = 8), classed as out of bounds by Affymetrix (n = 30), duplication or cryptic relatedness (identity by descent >0.185, n = 4), evidence of non-white European ancestry using PCA in conjunction with HapMap samples (n = 130; cut-off based on the minimum and maximum values of the top two principal components of the controls; Supplementary Figure 2). The details of all sample exclusions are provided in Supplementary Figure 3. We excluded SNPs from the analysis with: call rate <95%; different missing genotype rate between cases and controls at P < 10^−5^; MAF < 0.01; departure from Hardy–Weinberg equilibrium in controls at P < 10^−5^. The adequacy of the case–control matching and the possibility of differential genotyping of cases and controls were assessed using quantile-quantile (Q–Q) plots of test statistics.

### Published GWAS

### Statistical and bioinformatic analysis

Analyses were undertaken using R(v3.02)^24^ and PLINK^25^ software. The association between each SNP and the risk of CRC was assessed by the Cochran–Armitage trend test. ORs and associated 95% CIs were calculated by unconditional logistic regression. Phasing of GWAS SNP genotypes was performed using SHAPEIT(v2.644)^26^. Prediction of the untyped SNPs was carried out using IMPUTE(v2.3.0)^27^ based on the data from the 1000 Genomes Project (Phase 1 integrated variant set, v3.20101123)^28^ as reference. Imputed data were analyzed using SNPTEST(v2.4.1)^29^. Association meta-analyses only included markers with info scores >0.4, imputed call rates/SNP > 0.9 and MAFs > 0.01. The fidelity of imputation, as assessed by the concordance between imputed and sequenced SNPs, was examined in a subset of 200 UK cases. Meta-analyses were carried out using META(v2.4-1)^30^, under an inverse-weighted fixed-effects model using the genotype probabilities from IMPUTE, where a SNP was not directly typed. We calculated Cochran’s Q statistic to test for heterogeneity and the *I*^*2*^ statistic to quantify the proportion of the total variation that was caused by heterogeneity –*I*^*2*^ values ≥75% are considered characteristic of large heterogeneity^31^. Associations by sex, age and clinico-pathological phenotypes were examined by logistic regression in case-only analyses. The familial relative risk of CRC attributable to each variant was calculated as detailed by Pharoah *et al.*^32^ assuming the overall familial risk of CRC, as shown in epidemiological studies, is 2.2^33^.

To explore epigenetic profiles of association signals, we used ChromHMM^34^. States were inferred from ENCODE Histone Modification data on the CRC cell line HCT116 (DNAse, H3K4me3, H3K4me1, H3K27ac, Pol2 and CTCF)^35^ binarized using a multivariate Hidden Markov Model.

To examine whether any of the SNPs or their proxies (*i.e.* r^2^ > 0.8 in 1000genomes CEU reference panel) annotate putative transcription factor binding/enhancer elements we used the CADD (combined annotation dependent depletion) web-server^36^. We assessed sequence conservation using: PhastCons (<0.3 indicative of conservation), Genomic Evolutionary Rate Profiling^37^ (GERP) (−12 to 6, with 6 being indicative of complete conservation) and CADD (>10.0 deemed to be deleterious).

### Analysis of TCGA data

To examine for a relationship between SNP genotype and mRNA expression we made use of Tumor Cancer Genome Atlas (TCGA)^38^ RNA-seq expression and Affymetrix 6.0 SNP data (dbGaP accession number: phs000178.v7.p6) on 223 colorectal adenocarcinoma (COAD) and 75 rectal adenocarcinoma samples using a best proxy where SNPs were not represented directly. Association between normalised RNA counts per-gene and SNP genotype was quantified using the Kruskal-Wallis trend test. The frequency of somatic mutations in CRC was obtained using the CBioPortal for Cancer Genomics^39^,^40^ and TumorPortal web servers^41^.

### Pathway analysis

To determine whether any genes mapping to the three newly identified regions act in pathways already over-represented in GWAS regions we utilized the NCI pathway interaction database^42^. All genes within the LD block containing each tagSNP, or linked to the SNP through functional experiments (MYC) were submitted as a Batch query using the NCI-Nature curated data source.

### Assignment of microsatellite instability (MSI), KRAS, NRAS and BRAF status in cancers

Tumour MSI status in CRCs was determined using the mononucleotide microsatellite loci BAT25 and BAT26, which are highly sensitive MSI markers. Samples showing more than or equal to five novel alleles, when compared with normal DNA, at either or both markers were assigned as MSI-H (corresponding to MSI-high)^43^.

Tumours from the COIN study were screened for mutations in *KRAS* codons 12, 13, and 61 and *BRAF* codon 600 by pyrosequencing^18^. Additionally, *KRAS* (all three codons), *BRAF* (codons 594 and 600), and *NRAS* (codons 12 and 61) were screened for mutations by MALDI-TOF mass array (Sequenom, San Diego, CA, USA)^44^.

## Results

In the primary scan, 2,244 advanced (stage IV) CRC cases ascertained through the Medical Research Council (MRC) trials COIN^18^ and COIN-B^45^ were analysed with control data on 2,674 individuals from the WTCCC2 UK National Blood Service Control Group. After applying strict quality control criteria (Materials and Methods), we analysed 234,675 autosomal SNPs for association with CRC risk in 1,950 cases and 2,162 controls. A Q–Q plot of observed versus expected *χ*^2^-test statistics showed little evidence for an inflation of test statistics, thereby excluding the possibility of substantive hidden population substructure, cryptic relatedness among subjects or differential genotype calling (inflation factor *λ* = 1.05; Supplementary Figure 1).

We performed a meta-analysis of our primary scan data with five non-overlapping GWAS case-control series of Northern European ancestry, which have been previously reported (Supplementary Table 2). The adequacy of the case-control matching and possibility of differential genotyping of cases and controls was assessed using Q-Q plots of test statistics. λ_GC_ values^46^ for the UK1, Scotland1, VQ58, CCFR1 and CCFR2 studies were 1.02, 1.01, 1.01, 1.02 and 1.03 respectively (Supplementary Figure 1). Any ethnic outliers or individuals identified as related were excluded (Supplementary Figure 2).

After quality control procedures, the six GWAS provided data on 7,577 CRC cases and 9,979 controls. To maximise the prospects of identifying novel risk variants, we imputed over 10 million variants using 1000 Genomes Project Pilot data as a reference panel. Q-Q plots for all variants post-imputation did not show evidence of substantive over-dispersion introduced by imputation (Supplementary Figure 1).

### Meta-analysis

**Figure 1:**
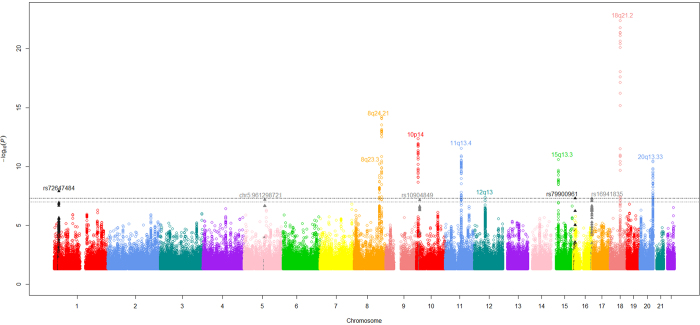
Genome-wide *P*-values (–log_10_*P*, *y*-axis) plotted against their respective chromosomal positions (*x*-axis). Known regions attaining genome-wide significance (*i.e*. *P* = 5.0 × 10^−8^) are labelled with their chromosomal location. Variants in grey lie in novel regions that reach the significance threshold level (*P* = 1.0 × 10^−7^) required for variants to be analysed further in this study. Variants in black lie in novel regions attaining genome-wide significance.

Associations for all 23 established European CRC risk SNPs showed a direction of effect consistent with previously reported studies, with eight of the loci having a *P*-value of <5.0 × 10^−8^ (Supplementary Table 3; Fig. 1). Additionally six SNPs previously identified in GWAS in Asian populations as determinants of CRC risk showed evidence for an association in this meta-analysis; albeit at varying degrees of significance (*P*-values ranging from 3.64 × 10^−2^ to 1.71 × 10^−3^; Supplementary Table 3); thereby providing support for trans-ethnic effects.

Excluding SNPs (including those correlated with *r*^2^ > 0.8) mapping to the risk loci, five variants in distinct regions of linkage disequilibrium (LD) were associated with CRC at *P* < 1.0 × 10^−7^ (Table 1; Fig. 1).

We assessed the fidelity of imputation in 200 UK cases by comparing imputed genotypes with those obtained by sequencing. For the three common variants (MAF > 0.05), rs72647484, rs16941835 and rs10904849 which each had imputation info scores >0.9 there was high correlation between imputed and directly typed genotype (r^2^ = 0.98, 1.00 and 0.99, respectively). For the rare variant rs79900961 (MAF = 0.016), the correlation was poor (r^2^ = 0.60). The call rate for the rare Indel on chromosome 5q15 (rs202110856) in the sequencing data was only 71% and both imputed heterozygotes were sequenced as homozygous reference. Therefore, only the three common variants at 1p36.12, 10p13 and 16q24.1 were subject to further analyses.

**Figure 2:**
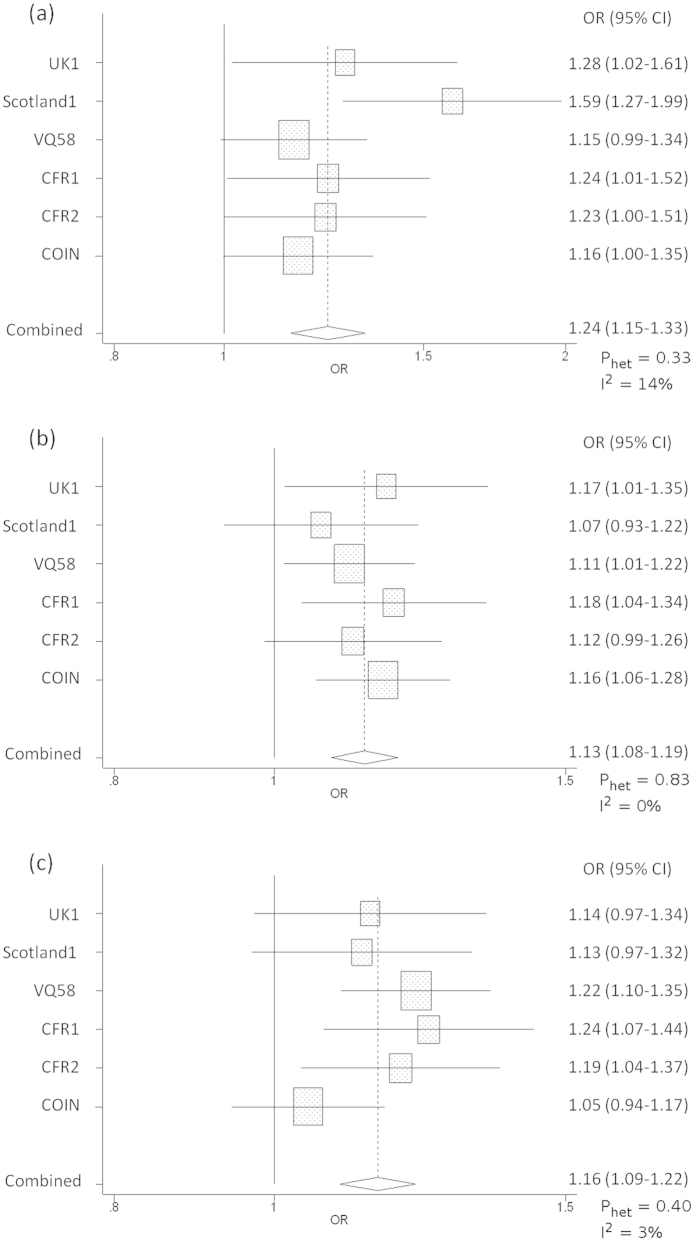
Forest plot of the odds ratios for the association between rs72647484, rs16941835, rs10904849 and CRC. Studies were weighted according to the inverse of the variance of the log of the OR calculated by unconditional logistic regression. *Horizontal lines:* 95% confidence intervals *(95% CI). Box:* OR point estimate; its area is proportional to the weight of the study. *Diamond (and broken line):* overall summary estimate, with confidence interval given by its width. *Unbroken vertical line:* null value (OR = 1.0).

**Figure 3:**
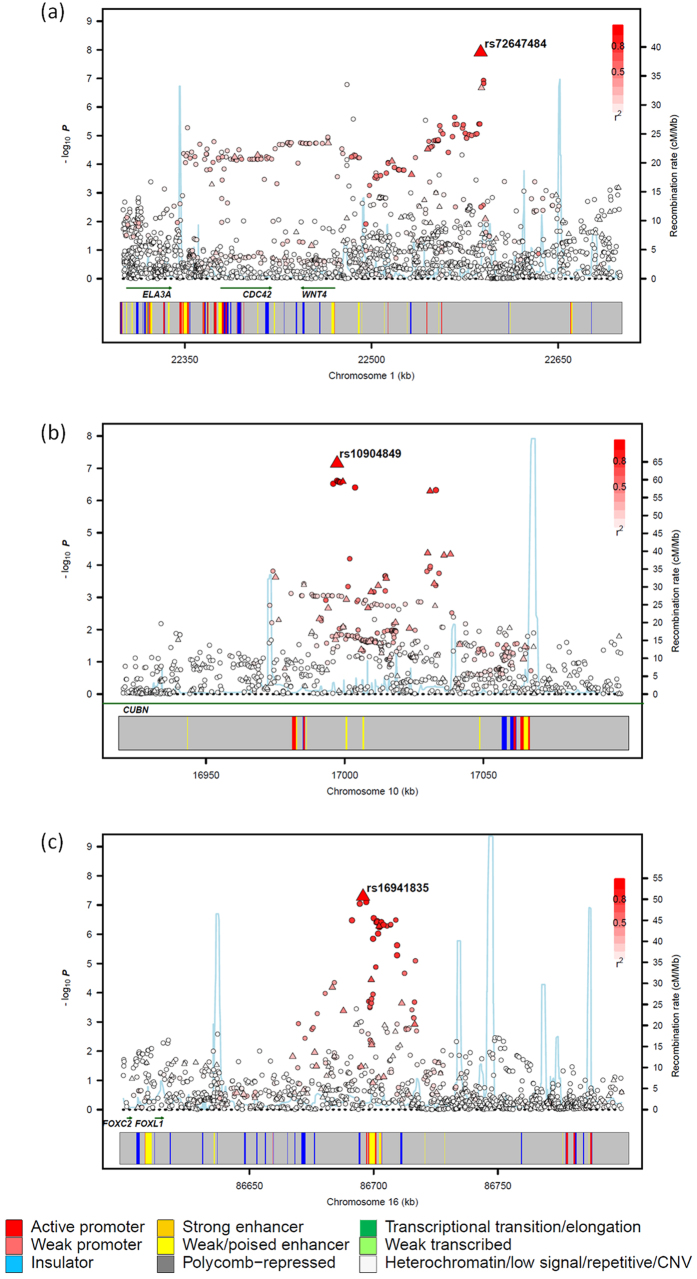
**Regional plot of association results and recombination rates for the (a) 1p36.12, (b) 10p13 and (c) 16q24.1 risk loci.** Association results of both genotyped (triangles) and imputed (circles) SNPs in the GWAS samples and recombination rates within the loci at 1p36.12 (**a**), 10p13 (**b**) and 16q24 (**c**). For each plot, −log_10_ *P* values (*y* axis) of the SNPs are shown according to their chromosomal positions (*x* axis). The top imputed SNP in each combined analysis is shown as a large triangle and is labelled by its rsID. The colour intensity of each symbol reflects the extent of LD with the top SNP: white (*r*^2^ = 0) through to dark red (*r*^2^ = 1.0), with *r*^2^ estimated from the 1000 Genomes Phase 1 data. Genetic recombination rates (cM/Mb), are shown with a light blue line. Physical positions are based on NCBI build 37 of the human genome. Also shown are the relative positions of genes and transcripts mapping to each region of association. The lower panel shows the chromatin state segmentation track (ChromHMM).

In the combined analysis of the six GWAS datasets, rs72647484, which maps to chromosome 1p36.12 (22,587,728 bps; NCBI build 37), showed the strongest evidence for association with CRC (*P* = 1.21 × 10^−8^; *P*_het_ = 0.33, *I*^2^ = 14%; Fig. 2a). rs72647484 maps within a 300 kb block of LD encompassing *WNT4* (wingless-type mmtv integration site family, member 4; MIM 603490) and *CDC42* (cell division cycle 42, MIM 116952; Fig. 3a). The second strongest association was provided by rs16941835 (*P* = 5.06 × 10^−8^; *P*_het_ = 0.40, *I*^2^ = 3%; Fig. 2c) which localises to the long non-coding RNA (lncRNA) RP11-58A18.1 at chromosome 16q24.1 (86,659,720 bps; NCBI build 37) within a 65 kb region of LD (Fig. 3c). The nearest coding gene, ~500 kb away, is the transcription factor *FOXL1.* The third strongest association was provided by rs10904849 (*P* = 7.01 × 10^−8^; *P*_het_ = 0.83, *I*^2^ = 0%; Fig. 2b) which localises to chromosome 10p13 (16,997,266 bps; NCBI build 37) within intron 31 of the gene encoding cubulin (*CUBN*; alias intrinsic factor-cobalamin receptor [IFCR], MIM 602997; Fig. 3b).

### Bioinformatic analysis of risk variants

To gain insight into the biological basis of the associations we analysed publicly available RNA-seq expression and SNP data from TCGA on 223 colonic and 75 rectal cancers using rs10904850 and rs2744753 as proxies for rs10904849 (r^2^ = 0.97; D’ = 1.00) and rs72647484 (r^2^ = 0.64; D’ = 0.89) respectively. After adjustment for multiple testing, no significant associations were seen between SNP genotype and expression of genes mapping to any of the three risk loci (Supplementary Table 4).

We examined whether any of the SNPs or their proxies (i.e. *r*^2^ > 0.8 in 1000 Genomes CEU reference panel) lie at putative transcription factor binding/enhancer elements and derived GERP and PhastCons scores to asses sequence conservation at these positions (Supplementary Table 5).

rs16941835 maps to a regulatory feature with histone modification suggestive of an enhancer element. rs10904852, in LD with rs10904849 (r^2^ = 0.95, *D*’ = 1.00) is conserved (GERP and PhastCons scores of 1.20 and 0.47 respectively) with CADD score of 11.53. A moderate CADD score (8.21) was associated with rs7267484 (22,590,125 bps) which is strong LD with rs72647489 (r^2^ = 0.93, *D*’ = 1.00). Six proxy SNPs in LD with rs16941835 showed some evidence of transcription factor binding (Supplementary Table 5). We made use of TCGA data to examine the frequency of somatic mutation of *CDC42*, *WNT4, FOXL1* or *CUBN* in CRC. None of these genes showed evidence of significant somatic mutation. Next, we conducted pathway analysis to determine whether any genes mapping to the three newly identified regions act in pathways already over-represented in GWAS. Pathways containing three or more genes are shown in Supplementary Table 6. While this analysis identifies the BMP-signalling pathway as expected, no catalogued pathways were discernible involving genes mapping to any of the newly identified regions.

It is increasingly recognized that some genetic variants can have pleiotropic effects, influencing the risk of more than one cancer type. To explore the possibility that rs72647484, rs10904849 or rs16941835 affects the risk of other malignancies, we examined the association with lung cancer^47^, acute lymphoblastic leukaemia^48^, multiple myeloma^49^, glioma^50^ and meningioma^51^ using data from previously reported GWASs. However, for these cancers, there was no evidence of rs72647484, rs10904849 or rs16941835 (or correlated SNP r^2^ ≥ 0.8) being associated with tumour risk (*i.e. P* > 0.05).

Finally, the relationship between clinico-pathological variables (sex, age at diagnosis, family history of CRC, tumour stage or microsatellite instability (MSI), KRAS-mutant status and BRAF-mutant status) and genotype at rs72647484, rs10904849 and rs16941835 was assessed by case-only logistic regression (Supplementary Table 7). There was evidence of a relationship between rs72647484 and *KRAS*-mutant status (*P* = 0.03) with the T risk allele associated with *KRAS*-mutant CRC; however this finding was not significant after accounting for multiple testing. None of the other SNPs showed any association with any of the clinico-pathological variables examined (*i.e. P* > 0.05).

## Discussion

We have provided evidence supporting the existence of new susceptibility loci for CRC at 1p36.12, 10p13 and 16q24.1. The 1p36.12 association implicates *WNT4* and/or *CDC42* as possible determinates of CRC risk. *WNT4* is part of a family of structurally related genes that encode cysteine-rich secreted glycoproteins that act as extracellular signalling factors. WNT4, WNT14, and WNT16 may play redundant roles in signalling through the CTNNB1-mediated canonical Wnt-pathway^52^ which is known to play a central role in colorectal tumorigenesis. Additionally, WNT4 signalling appears to play a pivotal role during organogenesis, acting as an autoinducer of mesenchyme-to-epithelial transition. Inactivating germline mutations in *WNT4* cause mullerian aplasia and hyperandrogenism (MIM 158330) and are responsible for the autosomal recessive SERKAL syndrome (Sex Reversal and Kidney, Adrenal, and Lung dysgenesis; MIM 611812). *A priori* dysfunction of either *WNT4* or *CDC42* could be the biological basis for the 1p36.12 association. Cdc42 is a Ras-related GTP-binding protein with roles in establishment of cell polarity, regulation of cell morphology, motility, and cell cycle progression in mammalian cells, and malignant transformation^53^. Notably, Cdc42 regulates the actin cytoskeleton through activation of WASP proteins and cell polarity through GSK3-beta and APC. Rho-GTPase signalling has a documented role in the development of CRC^54^. Activation of Rho GTPase Cdc42 promotes adhesion and invasion in CRC^55^ and targeting Cdc42 with AZA197 suppresses primary colon cancer growth and prolongs survival in a xenograft model through down regulation of *PAK1*^56^.

Since rs10904849 is intronic to *CUBN* and the region of LD does not encompass any other genes or transcripts, there is a high likelihood that the functional basis of the 10p13 association is mediated through *CUBN*. Cubilin is the intestinal receptor for the endocytosis of intrinsic factor-vitamin B12 and a receptor in epithelial apoA-I/HDL metabolism^57^. Additionally cubilin is an important co-receptor in the endocytic pathway for retrieval of 25(OH)D3-DBP complexes by megalin-mediated endocytosis in the kidney^58^. Germline mutations in *CUBN* cause recessive megaloblastic anemia-1 (MGA1; MIM 261100). It is conceivable that common genetic variance in CUBN, while being insufficient to cause a “MGA type phenotype” would have physiological effects by virtue of long term effect on the cellular bioavailability of B12. Although it is entirely speculative, as epidemiological studies have yet to convincingly establish levels of B12 as a risk factor for CRC^59^,^60^, its role in DNA biosynthesis makes genetically determined variation in B12 availability a plausible candidate for a role in the development of CRC.

LncRNAs are regulators of transcription and are increasingly recognised as playing a role in cancer biology. While there is currently no evidence to implicate the RP11-58A18.1 lncRNA in CRC, lncRNAs CCAT1 and CCAT2 probably do play such roles^61^,^62^, and it is entirely plausible that the impact of variation at 16q24.1 on risk is mediated through similar long range effects.

One of the reasons for the failure to identify these CRC-loci previously is that, in addition to the issue of study power, they were not optimally tagged by SNPs featured on many commercial arrays. The power of our study to detect the major common loci conferring risks of 1.2 or greater (such as the 18q24 variant) was high. Hence, it is very unlikely there are additional CRC SNPs with similar effects for alleles with frequencies >0.2 in populations of European ancestry.

In this study, we have only considered SNPs showing evidence of an association with a stipulated *P*-value threshold of <1 × 10^−7^. There exist, however, many variants with *P*-values just above this threshold which may also warrant investigation in a further study (Fig. 1). Hence further efforts to expand the scale of GWAS meta-analyses, in terms of both sample size and SNP coverage, and to increase the number of SNPs taken forward to large-scale replication, may identify additional variants for CRC.

In conclusion, we have provided evidence for 3 new susceptibility loci for CRC. Our data also provide further evidence for the value of meta-analysis and the value of imputation as a means of enhancing the detection of novel risk loci thereby extending the utility of GWAS data.

## Author Contributions

The study was designed and financial support was obtained by R.S.H., I.P.M.T., M.G.D. and J.P.C. The manuscript was drafted by R.S.H. with input from I.P.M.T., M.G.D. and J.P.C. All authors had access to data, analysis and had opportunity to contribute to drafting the manuscript. J.P.C. initiated and directed the GWAS of COIN and COIN-B. S.I. was responsible for COIN blood DNA extractions and quantification, R.H. for aliquoting and manifest preparation for Axiom genotyping and C.G.S. for somatic profiling of COIN tumours. N.A.A. and B.F.M. coordinated, and S.M.W. performed, the genotyping of COIN and COIN-B samples on the Axiom platform. T.S.M. was CI/co-CI of COIN and COIN-B, and R.K. provided access to linked clinico-pathological data. Cleaning of the COIN/COIN-B genotyping data and all statistical and bioinformatic analyses were conducted by N.W. and F.H., with contributions from S.D. and B.K., under the supervision of R.S.H. ICR - Sample preparation and genotyping were performed by A.L. and N.W. Oxford and local collaborators: subject recruitment and sample acquisition were done by E.B., M.G., L.M., R.K., D.K., and members of the CORGI Consortium. Sample preparation and genotyping were performed by C.P. Colon Cancer Genetics Group, Edinburgh and local collaborators: subject recruitment and sample acquisition were performed by S.M.F., C.H., H.C., I.D. and M.G.D., as well as members of SOCCS and COGS recruitment teams. Sample preparation was coordinated by S.M.F. Genotyping and analysis was performed and coordinated by S.M.F., C.H., M.G.D. and A.T. For the colon CFR datasets - D.D.B., A.K.W., J.H., M.J., F.S., G.C., S.G., N.L., P.N. and D.C. performed sample ascertainment and analysis.

## Additional Information

**How to cite this article**: Al-Tassan, N. A. *et al.* A new GWAS and meta-analysis with 1000Genomes imputation identifies novel risk variants for colorectal cancer. *Sci. Rep.* **5**, 10442; doi: 10.1038/srep10442 (2015).

## Supplementary Material

Supplementary Information

## Figures and Tables

**Table 1:** **Summary statistics for variants showing an association with CRC risk at** ***P*** **< 1.0 × 10** ^**−7**^.

							**Individual study** ***P*****-values**						**Meta-analysis**		
**Locus**	**Nearest gene(s)**	**SNP**	**Position (bps)**	**Alleles**	**RAF**	**INFO**	**UK1**	**Scotland1**	**VQ58**	**CFR1**	**CFR2**	**COIN**	**OR (95% CI)**	***P***	***P***_**het**_
1p36.12	*WNT4*/*CDC42*	rs72647484	22,587,728	TC	0.91	0.94 (0.85-0.99)	3.25 ×10^−2^	3.32 ×10^−5^	4.99 ×10^−2^	4.08 ×10^−2^	4.58 ×10^−2^	3.47 ×10^−2^	1.24 (1.15-1.33)	1.21 ×10^-8^	0.33
5q15	*ERAP1*	rs202110856	96,129,872	GGC	0.99	0.79 (0.66-0.92)	2.97 ×10^−1^	5.96 ×10^-8^	2.81 ×10^−2^	4.43 ×10^−1^	3.35 ×10^−1^	3.67 ×10^−1^	1.51 (1.23-1.86)	6.67 ×10^-8^	0.13
10p13	*CUBN*	rs10904849	16,997,266	GT	0.68	0.98 (0.97-1.00)	2.90 ×10^−2^	3.39 ×10^−1^	2.36 ×10^−2^	8.68 ×10^−3^	7.73 ×10^−2^	1.29 ×10^−3^	1.13 (1.08-1.19)	7.01×10^-8^	0.83
16p13.2	*C16orf72*	rs79900961	9,297,812	GA	0.98	0.70 (0.61-0.74)	2.21 ×10^−1^	8.68 ×10^−2^	1.04 ×10^−3^	2.54 ×10^−2^	2.41 ×10^−1^	1.02 ×10^−3^	1.49 (1.26-1.76)	4.93 ×10^-8^	0.76
16q24.1	*FOXL1*	rs16941835	86,695,720	GC	0.21	0.97 (0.92-0.99)	1.04 ×10^−1^	1.17 ×10^−1^	1.57 ×10^−4^	3.74 ×10^−3^	1.25 ×10^−2^	3.65 ×10^−1^	1.16 (1.09-1.22)	5.06 ×10^-8^	0.40

For each variant shown along with meta-analysis test statistics are the *P*-values from the six individual studies and imputation Information scores. Risk alleles are given in bold.

INFO, imputation Information score; P-het, P-value of heterogeneity between studies; RAF, risk allele frequency.

## References

[b1] Lichtenstein P. *et al.* Environmental and heritable factors in the causation of cancer--analyses of cohorts of twins from Sweden, Denmark, and Finland. N. Engl. J. Med. 343, 78–85 (2000). 10891514 10.1056/NEJM200007133430201

[b2] Aaltonen L., Johns L., Jarvinen H., Mecklin J. P. & Houlston R. Explaining the familial colorectal cancer risk associated with mismatch repair (MMR)-deficient and MMR-stable tumors. Clin. Cancer Res. 13, 356–361 (2007). 17200375 10.1158/1078-0432.CCR-06-1256

[b3] Lubbe S. J., Webb E. L., Chandler I. P. & Houlston R. S. Implications of familial colorectal cancer risk profiles and microsatellite instability status. J. Clin. Oncol. 27, 2238–2244 (2009). 19307499 10.1200/JCO.2008.20.3364

[b4] Broderick P. *et al.* A genome-wide association study shows that common alleles of SMAD7 influence colorectal cancer risk. Nat. Genet. 39, 1315–1317 (2007). 17934461 10.1038/ng.2007.18

[b5] Dunlop M. G. *et al.* Common variation near CDKN1A, POLD3 and SHROOM2 influences colorectal cancer risk. Nat. Genet. 44, 770–776 (2012). 22634755 10.1038/ng.2293 PMC4747430

[b6] Houlston R. S. *et al.* Meta-analysis of three genome-wide association studies identifies susceptibility loci for colorectal cancer at 1q41, 3q26.2, 12q13.13 and 20q13.33. Nat. Genet. 42, 973–977 (2010). 20972440 10.1038/ng.670 PMC5098601

[b7] Peters U. *et al.* Identification of Genetic Susceptibility Loci for Colorectal Tumors in a Genome-Wide Meta-analysis. Gastroenterology 144, 799–807 e24 (2013). 23266556 10.1053/j.gastro.2012.12.020 PMC3636812

[b8] Tenesa A. *et al.* Genome-wide association scan identifies a colorectal cancer susceptibility locus on 11q23 and replicates risk loci at 8q24 and 18q21. Nat. Genet. 40, 631–637 (2008). 18372901 10.1038/ng.133 PMC2778004

[b9] Tomlinson I. *et al.* A genome-wide association scan of tag SNPs identifies a susceptibility variant for colorectal cancer at 8q24.21. Nat. Genet. 39, 984–988 (2007). 17618284 10.1038/ng2085

[b10] Tomlinson I. P. *et al.* A genome-wide association study identifies colorectal cancer susceptibility loci on chromosomes 10p14 and 8q23.3. Nat. Genet. 40, 623–630 (2008). 18372905 10.1038/ng.111

[b11] Tomlinson I. P. *et al.* Multiple common susceptibility variants near BMP pathway loci GREM1, BMP4, and BMP2 explain part of the missing heritability of colorectal cancer. PLoS Genet. 7, e1002105 (2011). 21655089 10.1371/journal.pgen.1002105 PMC3107194

[b12] Whiffin N. *et al.* Identification of susceptibility loci for colorectal cancer in a genome-wide meta-analysis. Hum. Mol. Genet. 23, 4729–4737 (2014). 24737748 10.1093/hmg/ddu177 PMC4133584

[b13] Cui R. *et al.* Common variant in 6q26-q27 is associated with distal colon cancer in an Asian population. Gut 60, 799–805 (2011). 21242260 10.1136/gut.2010.215947 PMC3095478

[b14] Jia W. H. *et al.* Genome-wide association analyses in East Asians identify new susceptibility loci for colorectal cancer. Nat. Genet. 45, 191–196 (2013). 23263487 10.1038/ng.2505 PMC3679924

[b15] Wang H. *et al.* Trans-ethnic genome-wide association study of colorectal cancer identifies a new susceptibility locus in VTI1A. Nat. Commun. 5, 4613 (2014). 25105248 10.1038/ncomms5613 PMC4180879

[b16] Zhang B. *et al.* Large-scale genetic study in East Asians identifies six new loci associated with colorectal cancer risk. Nat. Genet. 46, 533–542 (2014). 24836286 10.1038/ng.2985 PMC4068797

[b17] The 1000 Genomes Project Consortium *et al.* An integrated map of genetic variation from 1,092 human genomes. Nature 491, 56–65 (2012). 23128226 10.1038/nature11632 PMC3498066

[b18] Maughan T. S. *et al.* Addition of cetuximab to oxaliplatin-based first-line combination chemotherapy for treatment of advanced colorectal cancer: results of the randomised phase 3 MRC COIN trial. Lancet 377, 2103–2114 (2011). 21641636 10.1016/S0140-6736(11)60613-2 PMC3159415

[b19] Midgley R. S. *et al.* Phase III randomized trial assessing rofecoxib in the adjuvant setting of colorectal cancer: final results of the VICTOR trial. J. Clin. Oncol. 28, 4575–4580 (2010). 20837956 10.1200/JCO.2010.29.6244

[b20] Power C. & Elliott J. Cohort profile: 1958 British birth cohort (National Child Development Study). Int. J. Epidemiol. 35, 34–41 (2006). 16155052 10.1093/ije/dyi183

[b21] Newcomb P. A. *et al.* Colon Cancer Family Registry: an international resource for studies of the genetic epidemiology of colon cancer. Cancer Epidemiol. Biomarkers Prev. 16, 2331–2343 (2007). 17982118 10.1158/1055-9965.EPI-07-0648

[b22] Hunter D. J. *et al.* A genome-wide association study identifies alleles in FGFR2 associated with risk of sporadic postmenopausal breast cancer. Nat. Genet. 39, 870–874 (2007). 17529973 10.1038/ng2075 PMC3493132

[b23] Yeager M. *et al.* Genome-wide association study of prostate cancer identifies a second risk locus at 8q24. Nat. Genet. 39, 645–649 (2007). 17401363 10.1038/ng2022

[b24] R Core Team 2013. R: A language and environment for statistical computing. . R Foundation for Statistical Computing, Vienna, Austria. URL http://www.R-project.org/ (Date of access 01/12/2014); (Accessed 01/12/2014).

[b25] Purcell S. *et al.* PLINK: a tool set for whole-genome association and population-based linkage analyses. Am J. Hum. Genet. 81, 559–575 (2007). 17701901 10.1086/519795 PMC1950838

[b26] Delaneau O., Marchini J. & Zagury J. F. A linear complexity phasing method for thousands of genomes. Nat. Methods 9, 179–181 (2012). 22138821 10.1038/nmeth.1785

[b27] Howie B. N., Donnelly P. & Marchini J. A flexible and accurate genotype imputation method for the next generation of genome-wide association studies. PLoS Genet 5, e1000529 (2009). 19543373 10.1371/journal.pgen.1000529 PMC2689936

[b28] 1000 Genomes. http://www.1000genomes.org/(Accessed 01/12/2014).

[b29] Marchini J., Howie B., Myers S., McVean G. & Donnelly P. A new multipoint method for genome-wide association studies by imputation of genotypes. Nat. Genet 39, 906–913 (2007). 17572673 10.1038/ng2088

[b30] Liu J. Z. *et al.* Meta-analysis and imputation refines the association of 15q25 with smoking quantity. Nat. Genet. 42, 436–440 (2010). 20418889 10.1038/ng.572 PMC3612983

[b31] Higgins J. P. & Thompson S. G. Quantifying heterogeneity in a meta-analysis. Stat. Med. 21, 1539–1558 (2002). 12111919 10.1002/sim.1186

[b32] Pharoah P. D., Antoniou A. C., Easton D. F. & Ponder B. A. Polygenes, risk prediction, and targeted prevention of breast cancer. N. Engl. J. Med. 358, 2796–2803 (2008). 18579814 10.1056/NEJMsa0708739

[b33] Johns L. E. & Houlston R. S. A systematic review and meta-analysis of familial colorectal cancer risk. Am. J. Gastroenterol. 96, 2992–3003 (2001). 11693338 10.1111/j.1572-0241.2001.04677.x

[b34] Ernst J. & Kellis M. Discovery and characterization of chromatin states for systematic annotation of the human genome. Nat. Biotechnol. 28, 817–825 (2010). 20657582 10.1038/nbt.1662 PMC2919626

[b35] The ENCODE Project: ENCyclopedia Of DNA Elements. http://www.genome.gov/encode/ (Accessed 01/12/2014).

[b36] Kircher M. *et al.* A general framework for estimating the relative pathogenicity of human genetic variants. Nat. Genet. 46, 310–315 (2014). 24487276 10.1038/ng.2892 PMC3992975

[b37] Cooper G. M. *et al.* Distribution and intensity of constraint in mammalian genomic sequence. Genome. Res. 15, 901–913 (2005). 15965027 10.1101/gr.3577405 PMC1172034

[b38] The Cancer Genome Atlas http://cancergenome.nih.gov/ (Accessed 01/12/2014).

[b39] Gao J. *et al.* Integrative analysis of complex cancer genomics and clinical profiles using the cBioPortal. Sci. Signal 6, pl1 (2013). 10.1126/scisignal.2004088 PMC4160307 23550210

[b40] Cerami E. *et al.* The cBio cancer genomics portal: an open platform for exploring multidimensional cancer genomics data. Cancer Discov. 2, 401–404 (2012). 22588877 10.1158/2159-8290.CD-12-0095 PMC3956037

[b41] Lawrence M. S. *et al.* Discovery and saturation analysis of cancer genes across 21 tumour types. Nature 505, 495–501 (2014). 24390350 10.1038/nature12912 PMC4048962

[b42] NCI pathway interaction database. Accessed 01/12/2014. http://pid.nci.nih.gov/ (2014).

[b43] Boland C. R. *et al.* A National Cancer Institute Workshop on Microsatellite Instability for cancer detection and familial predisposition: development of international criteria for the determination of microsatellite instability in colorectal cancer. Cancer Res. 58, 5248–5257 (1998). 9823339

[b44] Smith C. G. *et al.* Somatic profiling of the epidermal growth factor receptor pathway in tumors from patients with advanced colorectal cancer treated with chemotherapy +/- cetuximab. Clin. Cancer Res. 19, 4104–4113 (2013). 23741067 10.1158/1078-0432.CCR-12-2581 PMC3732482

[b45] Wasan H. *et al.* Intermittent chemotherapy plus either intermittent or continuous cetuximab for first-line treatment of patients with KRAS wild-type advanced colorectal cancer (COIN-B): a randomised phase 2 trial. Lancet Oncol. 15, 631–639 (2014). 24703531 10.1016/S1470-2045(14)70106-8 PMC4012566

[b46] Clayton D. G. *et al.* Population structure, differential bias and genomic control in a large-scale, case-control association study. Nat. Genet. 37, 1243–1246 (2005). 16228001 10.1038/ng1653

[b47] Broderick P. *et al.* Deciphering the impact of common genetic variation on lung cancer risk: a genome-wide association study. Cancer. Res. 69, 6633–6641 (2009). 19654303 10.1158/0008-5472.CAN-09-0680 PMC2754318

[b48] Migliorini G. *et al.* Variation at 10p12.2 and 10p14 influences risk of childhood B-cell acute lymphoblastic leukemia and phenotype. Blood 122, 3298–3307 (2013). 23996088 10.1182/blood-2013-03-491316

[b49] Chubb D. *et al.* Common variation at 3q26.2, 6p21.33, 17p11.2 and 22q13.1 influences multiple myeloma risk. Nat. Genet. 45, 1221–1225 (2013). 23955597 10.1038/ng.2733 PMC5053356

[b50] Sanson M. *et al.* Chromosome 7p11.2 (EGFR) variation influences glioma risk. Hum. Mol. Genet. 20, 2897–2904 (2011). 21531791 10.1093/hmg/ddr192 PMC3118762

[b51] Dobbins S. E. *et al.* Common variation at 10p12.31 near MLLT10 influences meningioma risk. Nat. Genet. 43, 825–827 (2011). 21804547 10.1038/ng.879 PMC5053355

[b52] Guo X. *et al.* Wnt/beta-catenin signaling is sufficient and necessary for synovial joint formation. Genes Dev. 18, 2404–2417 (2004). 15371327 10.1101/gad.1230704 PMC522990

[b53] Wu W. J., Erickson J. W., Lin R. & Cerione R. A. The gamma-subunit of the coatomer complex binds Cdc42 to mediate transformation. Nature 405, 800–804 (2000). 10866202 10.1038/35015585

[b54] Leve F. & Morgado-Diaz J. A. Rho GTPase signaling in the development of colorectal cancer. J. Cell Biochem. 113, 2549–2559 (2012). 22467564 10.1002/jcb.24153

[b55] Gao L., Bai L. & Nan Q. Activation of Rho GTPase Cdc42 promotes adhesion and invasion in colorectal cancer cells. Med. Sci. Monit Basic Res. 19, 201–207 (2013). 23884297 10.12659/MSMBR.883983 PMC3735386

[b56] Zins K., Gunawardhana S., Lucas T., Abraham D. & Aharinejad S. Targeting Cdc42 with the small molecule drug AZA197 suppresses primary colon cancer growth and prolongs survival in a preclinical mouse xenograft model by downregulation of PAK1 activity. J. Transl. Med. 11, 295 (2013). 24279335 10.1186/1479-5876-11-295 PMC4222769

[b57] Kozyraki R. *et al.* The human intrinsic factor-vitamin B12 receptor, cubilin: molecular characterization and chromosomal mapping of the gene to 10p within the autosomal recessive megaloblastic anemia (MGA1) region. Blood 91, 3593–3600 (1998). 9572993

[b58] Nykjaer A. *et al.* Cubilin dysfunction causes abnormal metabolism of the steroid hormone 25(OH) vitamin D(3). Proc. Natl. Acad. Sci. U S A 98, 13895–13900 (2001). 11717447 10.1073/pnas.241516998 PMC61138

[b59] Bassett J. K. *et al.* Dietary intake of B vitamins and methionine and colorectal cancer risk. Nutr. Cancer 65, 659–667 (2013). 23859033 10.1080/01635581.2013.789114

[b60] Razzak A. A. *et al.* Associations between intake of folate and related micronutrients with molecularly defined colorectal cancer risks in the Iowa Women’s Health Study. Nutr. Cancer 64, 899–910 (2012). 23061900 10.1080/01635581.2012.714833 PMC3584680

[b61] Zhai H. *et al.* Clinical significance of long intergenic noncoding RNA-p21 in colorectal cancer. Clin. Colorectal. Cancer 12, 261–266 (2013). 24012455 10.1016/j.clcc.2013.06.003

[b62] Ling H. *et al.* CCAT2, a novel noncoding RNA mapping to 8q24, underlies metastatic progression and chromosomal instability in colon cancer. Genome Res. 23, 1446–1461 (2013). 23796952 10.1101/gr.152942.112 PMC3759721

