# Supplementary material for: A new GWAS and meta-analysis with 1000Genomes imputation identifies novel risk variants for colorectal cancer
Source: Sci Rep. 2015 May 20;5:10442. doi: 10.1038/srep10442 (PMC4438486; doi:10.1038/srep10442)
Supplement: Supplementary Information [file srep10442-s1.pdf]

SUPPLEMENTARY MATERIAL

A new GWAS and meta-analysis with 1000Genomes imputation identifies novel risk variants for colorectal cancer

Nada A. Al-Tassan[1†], Nicola Whiffin[2†], Fay J. Hosking[2†], Claire Palles[3], Philip Law[1], Susan M. Farrington[4], Sara E. Dobbins[2], Rebecca Harris[5], Maggie Gorman[3], Albert Tenesa[4,6], Brian F. Meyer[1], Salma M. Wakil[1], Ben Kinnersley[2], Harry Campbell[7], Lynn Martin[3], Christopher G. Smith[5], Shelley Idziaszczyk[5], Ella Barclay[3], Timothy S. Maughan[8], Richard Kaplan[9], Rachel Kerr[10], David Kerr[11], Daniel D. Buchannan[12,13], Aung Ko Win[13], John Hopper[13], Mark Jenkins[13], Noralane M. Lindor[14], Polly A. Newcomb[15], Steve Gallinger[16], David Conti[17], Fred Schumacher[17], Graham Casey[17], Malcolm G. Dunlop[4‡], Ian P. Tomlinson[3‡], Jeremy P. Cheadle[5‡] and Richard S Houlston[2‡*]

SUPPLEMENTARY METHODS

Ethics statement

Collection of blood samples and clinico-pathological information from all subjects was undertaken with informed consent and ethical review board approval in accordance with the tenets of the Declaration of Helsinki.

Subjects and datasets

In all cases CRC was defined according to the 9th revision of the International Classification of Diseases (ICD) by codes 153–154.

COIN

The COIN GWAS was based on 2,244 CRC cases (64% male, mean age 61 years, SD=10) ascertained through two independent Medical Research Council clinical trials of advanced/metastatic CRC; COIN and COIN-B (9). COIN patients were randomised 1:1:1 to receive continuous oxaliplatin and fluoropyrimidine chemotherapy, continuous chemotherapy plus cetuximab, or intermittent chemotherapy. COIN-B patients were randomised 1:1 to receive intermittent chemotherapy and cetuximab or intermittent chemotherapy and continuous cetuximab. All patients gave informed consent for their samples to be used for bowel cancer research (approved by REC [04/MRE06/60]).

DNA was extracted from EDTA-venous blood samples using conventional methods and quantified using Nanodrop spectrophotometry (Thermo Scientific, MA, USA). Cases were genotyped using Affymetrix Axiom Arrays according to the manufacturer's recommendations (Affymetrix, Santa Clara, CA 95051, USA) at the King Faisal Specialist Hospital and Research Center, Saudi Arabia (under IRB approval 2110033). Genotyping quality control was tested using duplicate DNA samples, together with direct sequencing of significantly associated SNPs in a subset of samples to confirm genotyping accuracy. For all SNPs, >99% concordant results were obtained.

For controls, we made use of publicly accessible Affymetrix 6.0 array data generated by the Wellcome Trust Case Control Consortium 2 (WTCCC2) on 2,674 individuals from the UK Blood Service Control Group. We excluded individuals from analysis if they failed one or more of the following thresholds: overall successfully genotyped SNPs < 95% (n = 122), discordant sex information (n = 8), classed as out of bounds by Affymetrix (n = 30), duplication or cryptic relatedness (identity by descent >0.185, n = 4), and evidence of non-

white European ancestry by PCA-based analysis in comparison with HapMap samples (n = 130; cut-off based on the minimum and maximum values of the top two principal components of the controls; Supplementary Figure 2). We excluded SNPs from analysis if they failed one or more of the following thresholds: call rate <95%; different missing genotype rate between cases and controls at $P<10^{-5}$; MAF <0.01; departure from Hardy–Weinberg equilibrium in controls at $P<10^{-5}$. The details of all sample exclusions are provided in Supplementary Figure 3. The adequacy of the case–control matching and the possibility of differential genotyping of cases and controls were assessed using Q–Q plots of test statistics. The inflation factor λ_{GC} was calculated by dividing the median of the lower 90% of the test statistics by the median of the lower 90% of the expected values from a $\chi 2$ distribution with 1 d.f.

Published GWAS

UK1 (CORGI) (7) comprised 940 cases with colorectal neoplasia (47% male) ascertained through the Colorectal Tumour Gene Identification (CoRGI) consortium. All had at least one first-degree relative affected by CRC and one or more of the following phenotypes: CRC at age 75 or less; any colorectal adenoma (CRAd) at age 45 or less; ≥3 colorectal adenomas at age 75 or less; or a large (>1 cm diameter) or aggressive (villous and/or severely dysplastic) adenoma at age 75 or less. The 965 controls (45% male) were spouses or partners unaffected by cancer and without a personal family history (to second degree relative level) of colorectal neoplasia. Known dominant polyposis syndromes, HNPCC/Lynch syndrome or bi-allelic MUTYH mutation carriers were excluded. All cases and controls were of white UK ethnic origin.

Scotland1 (COGS) (7) included 1,012 CRC cases (51% male; mean age at diagnosis 49.6 years, SD ± 6.1) and 1,012 cancer-free population controls (51% male; mean age 51.0 years; SD ± 5.9). Cases were selected for early age at onset (age ≤55 years). Known dominant polyposis syndromes, HNPCC/Lynch syndrome or bi-allelic MUTYH mutation carriers were excluded. Control subjects were sampled from the Scottish population NHS registers, matched by age (±5 years), gender and area of residence within Scotland.

VQ58 comprised 1800 CRC cases (1,099 males, mean age of diagnosis 62.5 years; SD ± 10.9) from the VICTOR (33) and QUASAR2 (www.octooxford.org.uk/alltrials/trials/q2.html) trials and 2,690 population control genotypes (1,391 male) from the Wellcome Trust Case–Control Consortium 2 (WTCCC2) 1958 birth cohort (11) (also known as the National Child Development Study), which included all births in England, Wales and Scotland during a single week in 1958.

The CCFR1 data set comprised 1,290 familial CRC cases and 1,055 controls from the Colon Cancer Family Registry (http://coloncfr.org) (12). The cases were recently diagnosed CRC

cases reported to population complete cancer registries in the USA (Puget Sound, Washington State) were recruited by the Seattle Familial Colorectal Cancer Registry; in Canada (Ontario) who were recruited by the Ontario Familial Cancer Registry; and in Australia (Melbourne, Victoria) who were recruited by the Australasian Colorectal Cancer Family Study. Controls were population-based and for this analysis were restricted to those without a family history of CRC. The CCFR2 data set comprised a further 796 cases from the Colon Cancer Family Registry, recruited from centres in Australia, Ontario, Seattle, USC, Mayo and Hawaii, and 2,236 controls from the Cancer Genetic Markers of Susceptibility studies of breast (n=1,142) and prostate (n = 1,094) cancer (13, 14) . All subjects included in CCFR1 and CCFR2 were non-Hispanic white.

The VQ, UK1 and Scotland1 GWA cohorts were genotyped using Illumina Hap300, Hap240S, Hap370, Hap550 or Omni2.5M arrays. 1958BC genotyping was performed as part of the WTCCC2 study on Hap1.2M-Duo Custom arrays. The CCFR samples were genotyped using Illumina Hap1M, Hap1M-Duo or Omni-express arrays. CGEMS samples were genotyped using Illumina Hap300 and Hap240 or Hap550 arrays. After applying the same quality control as that performed for COIN and COIN-B, data on 7,577 CRC cases and 9,979 controls were available for the meta-analysis (Supplementary Figure 1).

Statistical and bioinformatic analysis

Analyses were primarily undertaken using R (v3.02) and SNPTEST (v2.4.1) and PLINK(34) software. The association between each SNP and the risk of CRC was assessed by the Cochran–Armitage trend test. ORs and associated 95% CIs were calculated by unconditional logistic regression. Phasing of GWAS SNP genotypes was performed using SHAPEIT v2.644. Prediction of the untyped SNPs was carried out using IMPUTEv2 (v2.3.0) based on the data from the 1000 Genomes Project (Phase 1 integrated variant set, v3.20101123, released on the IMPUTEv2 website on 9 December 2013) as reference. Imputed data were analysed using SNPTEST v2.4.1 to account for uncertainties in SNP prediction. Association meta-analyses only included markers with info scores >0.4, imputed call rates/SNP >0.9 and MAFs >0.01. The fidelity of imputation, as assessed by the concordance between imputed and sequenced SNPs, was examined in a subset of 200 UK cases.

Meta-analyses were carried out using META v2.4-1, using the genotype probabilities from IMPUTEv2, where a SNP was not directly typed. We calculated Cochran's Q statistic to test for heterogeneity and the I^2 statistic to quantify the proportion of the total variation that was caused by heterogeneity (35). I^2 values ≥75% are considered characteristic of large heterogeneity (35-37). Associations by sex, age and clinico-pathological phenotypes were examined by logistic regression in case-only analyses. LD blocks were defined on the basis of HapMap recombination rate (cM/Mb) as defined using the Oxford recombination hotspots and on the basis of the distribution of CIs defined by Gabriel et al (37).

The familial relative risk of CRC attributable to a variant was calculated using the formula (38):

$$\lambda^* = \frac{p(pr_2 + qr_1)^2 + q(pr_1 + q)^2}{(p^2 r_2 + 2pqr_1 + q^2)^2}$$

where p is the population frequency of the minor allele, q=1–p, and r_1 and r_2 are the relative risks (approximated by ORs) for heterozygotes and the rarer homozygotes relative to the more common homozygotes respectively. From λ^*, it is possible to quantify the influence of the locus on the overall familial risk of CRC in first-degree relatives of CRC patients. Assuming a multiplicative interaction between risk alleles, the proportion of the overall familial risk attributable to the locus is given by log (λ^*)/log(λ_0), where λ_0, the overall familial risk of CRC, shown in epidemiological studies is 2.2 (39).

To explore epigenetic profiles of association signals, we used ChromHMM (40). States were inferred from ENCODE Histone Modification data on the CRC cell line HCT116 (DNAse, H3K4me3, H3K4me1, H3K27ac, Pol2 and CTCF) binarized using a multivariate Hidden Markov Model.

To examine whether any of the SNPs or their proxies (i.e. r2 > 0.8 in 1000genomes CEU reference panel) annotate putative transcription factor binding/enhancer elements we used the CADD (combined annotation dependent depletion) webserver (16) which integrates information from the Ensembl Variant Effect Predictor (VEP) (41) and ENCODE (42). We assessed sequence conservation using PhastCons (score <0.3 indicative of conservation) and Genomic Evolutionary Rate Profiling (GERP). GERP scores (−12 to 6, with 6 being indicative of complete conservation) reflect the proportion of substitutions at that site rejected by selection compared with observed substitutions expected under a neutral evolutionary model, based on sequence alignment of 34 mammalian species (43). We also derived CADD scores to assess functionality of non-coding changes (CADD score >10.0 deemed to be deleterious).

Analysis of TCGA data

Relationship between SNP genotype and mRNA expression.

To examine for a relationship between SNP genotype and mRNA expression we made use of Tumor Cancer Genome Atlas (TCGA) RNA-seq expression and Affymetrix 6.0 SNP data (dbGaP accession number: phs000178.v7.p6) on 223 colorectal adenocarcinoma (COAD) and 75 rectal adenocarcinoma samples using a best proxy where SNPs were not represented directly. Association between normalised RNA counts per-gene and SNP genotype was quantified using the Kruskal-Wallis trend test.

Mutation frequency.

The frequency of somatic mutations in CRC was obtained using the CBioPortal for Cancer Genomics and TumorPortal web servers.

Pathway analysis

To determine whether any genes mapping to the three newly identified regions act in pathways already over-represented in GWAS regions we utilized the NCI pathway interaction database (http://pid.nci.nih.gov/index.shtml). All genes within the LD block containing each tagSNP, or linked to the SNP through functional experiments (MYC) were submitted as a Batch query using the NCI-Nature curated data source.

Assignment of microsatellite instability (MSI), KRAS, NRAS and BRAF status in cancers

Tumour MSI status in CRCs was determined using the mononucleotide microsatellite loci BAT25 and BAT26, which are highly sensitive MSI markers. Briefly, 10 µm sections were cut from formalin-fixed paraffin-embedded CRC tumours, lightly stained with toluidine blue and regions containing at least 60% tumour microdissected. Tumour DNA was extracted using the QIAamp DNA Mini kit (Qiagen, Crawley, UK) according to the manufacturer's instructions and genotyped for the BAT25 and BAT26 loci using 32P–labelled oligonucleotide primers or FAM-labelled BAT26 and HEX-labelled BAT25 primers with visualisation on an ABI 3100 (Life Technologies, CA, USA). Samples showing more than or equal to five novel alleles, when compared with normal DNA, at either or both markers were assigned as MSI-H (corresponding to MSI-high)(44).

Tumours from the COIN study were screened for mutations in KRAS codons 12, 13, and 61 and BRAF codon 600 by pyrosequencing (9). Additionally, KRAS (all three codons), BRAF (codons 594 and 600), and NRAS (codons 12 and 61) were screened for mutations by MALDI-TOF mass array (Sequenom, San Diego, CA, USA) (45).

SUPPLEMENTARY TABLE AND FIGURE LEGENDS

Supplementary Table 1: Individual variance in risk associated with colorectal cancer SNPs

Supplementary Table 2: Summary of the sample sets used in the study. The numbers shown are after stringent QC measures (Supplementary Figure 3).

Supplementary Table 3: Best association signals from previously published risk loci

(A): Previously published risk loci discovered in European populations. Shown for each region are the GWAS tagSNP, the most associated variant within a 500kb window in the imputation and the associated odds ratio and P-values associated with each along with the linkage disequilibrium metrics between the SNPs. Imputation was not carried out on the X chromosome so this locus was not included. Genes with an asterisk (*) were also found to be significant in East Asian populations.

(B): Previously published risk loci discovered in East Asian populations. Shown for each region are the GWAS SNPs with the associated odds ratio and P-values for both the reported study and this study. RAF in the EUR population were obtained from the 1000 Genomes Project, and are shown in parentheses.

Supplementary Table 4: Relationship between SNP genotype and gene expression from RNA-seq data in 223 colon and 75 rectal cancers.

Supplementary Table 5: Summary of genomic annotation by CADD. Data are shown for rs72647484, rs16941835 and rs10904849 (in red) and proxy SNPs ($r2>0.8$ in 1000 Genomes

phase 1 data) demonstrating evidence of histone marks, transcription factor occupancy and evolutionary conservation (GERP and PhastCons). Also indicated are RegulomeDB and CADD scores.

Supplementary Table 6: Pathways overrepresented in GWAS regions. All genes within the LD block containing each tagSNP, or linked to the SNP through functional experiments, were uploaded to the NCI pathway interaction database. Displayed are pathways containing three or more genes.

Supplementary Table 7: Relationship between SNP genotype and sex, age at diagnosis of CRC, tumor site (rectal [ICD9:154], colonic [ICD9:153]), stage, family history of CRC (≥1 affected first degree relative), MSI status and both KRAS and BRAF mutant status.

Supplementary Figure 1: Quantile-Quantile (Q-Q) plots of observed and expected χ^2 values of association between SNP genotype and colorectal cancer risk. (a) UK1, (b) Scotland1, (c) VQ58, (d) CCFR1, (e) CCFR2, (f) COIN, (g) UK1 after imputation, (h) Scotland1 after imputation, (i) VQ58 after imputation, (j) CCFR1 after imputation, (k) CCFR2 after imputation, (l) COIN after imputation and (m) Meta-analysis.

Supplementary Figure 2: **Identification of individuals of non-European ancestry in cases and controls.** The first two principal components of the analysis are plotted. (a) UK1, (b) Scotland1, (c) VQ58, (d) CCFR1, (e) CCFR2 and (f) COIN. HapMap CEU individuals are plotted in blue; CHB+JPT individuals are plotted in green; YRI individuals are plotted in red; GWAS cases are plotted as circles and controls as triangles.

Supplementary Figure 3: Details of the quality control filters applied to each GWAS. Samples were excluded due to call rate (<95% or failed genotyping), Ethnicity (principle components analysis or other samples reported to be not of white, European descent), Relatedness (any individuals found to be duplicated or related within or between data sets through IBS), sex discrepancies or others (cases found to contain a previously reported susceptibility allele, controls with a 1st degree relative with CRC, low concordance of genotyping in duplicates or samples which have been subsequently withdrawn from a study).

Supplementary Table 1: Individual variance in risk associated with colorectal cancer SNPs

Locus	dbSNP No.	Gene	Risk-Allele Frequency	Relative Risk per Allele	% of Total Variance in Risk Explained‡	Reference
1q25.3	rs10911251	LAMC1	0.57	1.05	0.08	1
1q41	rs6691170	DUSP10	0.34	1.08	0.2	2
3q26.2	rs10936599	TERC, MYNN	0.76	1.08	0.17	2
6p21.31	rs1321311	CDKN1A	0.23	1.11	0.26	3
8q23.3	rs16892766	EIF3H	0.08	1.23	0.43	4
8q24.21	rs6983267	MYC	0.49	1.18	0.91	5
10p14	rs10795668	GATA3	0.66	1.15	0.63	4
10q24.2	rs1035209	SLC25A28, NKX2-3	0.19	1.15	0.39	6
11q13.4	rs3824999	POLD3	0.5	1.15	0.65	3
11q23.1	rs3802842	FLJ45803	0.68	1.14	0.48	7
12p13.32	rs3217810	CCND2	0.12	1.07	0.07	1
12q13.13	rs11169552	DIP2B, ATF1	0.74	1.1	0.23	2
14q22.2	rs4444235	BMP4	0.45	1.1	0.32	8
15q14	rs4779584	GREM1, SCG5	0.82	1.18	0.56	4
16q22.1	rs9929218	CDH1	0.69	1.09	0.2	8
18q21.2	rs4939827	SMAD7	0.52	1.22	1.3	9
19q12	rs10411210	RHPN2, GPATCH1	0.91	1.12	0.14	8
20p12.3	rs961253	BMP2	0.37	1.11	0.36	8
20p12.3	rs4813802	BMP2	0.36	1.11	0.33	10
20q13.33	rs4925386	LAMA5	0.31	1.1	0.27	2
					7.98	

For a single allele (*i*) of frequency *p*, relative risk *R* and log risk *r*, the variance (V_i) of the risk distribution due to that allele is given by

$$V_i = (1-p)^2E^2 + 2p(1-p)(r-E)^2 + p^2(2r-E)^2$$

Where *E* is the expected value of *r* given by

$$E = 2p(1-p)r + 2p^2r$$

For multiple risk alleles the distribution of risk in the population tends towards the normal with variance

$$V = \sum V_i$$

The total genetic variance (*V*) for all susceptibility alleles has been estimated to be √2.2. Thus the fraction of the genetic risk explained by a single allele is given by

$$V_i/V$$

‡ Method from Pharoah P *et al* N Eng J Med 2008; 358:2796-803. Familial risk of CRC assumed to be 2.2 (Johns LE, Houlston RS. Am J Gastroenterol. 2001; 96:2992-03).

REFERENCES

1. Peters et al. *Identification of Genetic Susceptibility Loci for Colorectal Tumors in a Genome-Wide Meta-analysis.* Gastroenterology, 2013. **144**(4): p. 799-807
2. Houlston et al. *Meta-analysis of three genome-wide association studies identifies susceptibility loci for colorectal cancer at 1q41, 3q26.2, 12q13.13 and 20q13.33.* Nat Gen, 2010. **42**(11): p. 973-7
3. Dunlop et al. *Common variation near CDKN1A, POLD3 and SHROOM2 influences colorectal cancer risk.* Nat Gen, 2012. **44**(7): p. 770-6
4. Tomlinson et al. *A genome-wide association study identifies colorectal cancer susceptibility loci on chromosomes 10p14 and 8q23.3.* Nat Gen, 2008. **40**(5): p. 623-30
5. Tomlinson et al. *A genome-wide association scan of tag SNPs identifies a susceptibility variant for colorectal cancer at 8q24.21.* Nat Gen, 2007. **39**(8): p. 984-8
6. Whiffin et al. *Identification of susceptibility loci for colorectal cancer in a genome-wide meta-analysis.* Hum Mol Genet, 2014. **23**(17): p. 4729-37
7. Tenesa et al. *Genome-wide association scan identifies a colorectal cancer susceptibility locus on 11q23 and replicates risk loci at 8q24 and 18q21.* Nat Gen, 2008. **40**(5): p. 631-7
8. Houlston et al. *Meta-analysis of genome-wide association data identifies four new susceptibility loci for colorectal cancer.* Nat Gen, 2008. **40**(12): p. 1426-35
9. Broderick et al*. A genome-wide association study shows that common alleles of SMAD7 influence colorectal cancer risk.* Nat Gen, 2007. **39**(11): p. 1315-7
10. Tomlinson et al. *Multiple Common Susceptibility Variants near BMP Pathway Loci GREM1, BMP4, and BMP2 Explain Part of the Missing Heritability of Colorectal Cancer.* PLoS Genetics, 2011. **7**(6)

Supplementary Table 2: Summary of the sample sets used in the study. The numbers shown are after stringent QC measures (Supplementary Figure 3).

Study	Cases	Controls	GWAS Chip	Inflation factor (λ)*	
				Before Imputation	After imputation
**UK1**	890	900	Illumina Hap550	1.02	1.03
**Scotland1**	973	998	Illumina Hap300/240S	1.01	1.04
**VQ58**	1,794	2,686	Illumina Hap300/370, Illumina 1M	1.01	1.04
**CCFR1**	1,175	999	Illumina 1M, 1M Duo	1.02	1.03
**CCFR2**	795	2,234	Illumina 1M, Omni express	1.03	1.08
**COIN**	1,950	2,162	Affymetrix Axiom	1.05	1.10
**Overall**	7,577	9,979	-	1.03	1.07

*Inflation factors (λ) were calculated by dividing the mean of the lower 90% of the test statistics by the mean of the lower 90% of the expected values from a χ^2 distribution with 1 d.f. Only SNPs with minor allele frequency > 0.05, imputation INFO >0.4, *P*-heterogeneity>0.01 and *P*-HWE>0.01 were considered in calculations.

Supplementary Table 3: Best association signals from previously published risk loci.

(A): Previously published risk loci discovered in European populations. Shown for each region are the GWAS tagSNP, the most associated variant in the imputation and the associated odds ratio and *P*-values associated with each along with the linkage disequilibrium metrics between the SNPs. Imputation was not carried out on the X chromosome so this locus was not included. Genes with an asterisk (*) were also found to be significant in East Asian populations.

Locus	Nearest Gene(s)	GWAS tagSNP	Lead variant this study	Location (bp)	r2 with tagSNP	D' with tagSNP	Risk Allele	Alt Allele	RAF	metaOR	meta*P*	Reference
**1q25.3**	*LAMC1 **	rs10911251		183,081,194			A	C	0.54	1.09	1.75×10^{-2}	1
			rs78164010	183,117,398	0.08	-	A	T	0.01	1.34	7.43×10^{-5}	
**1q41**	*DUSP10 **	rs6691170		222,045,446			T	G	0.40	1.08	1.05×10^{-4}	2
			rs11118883	222,061,022	0.40	0.75	A	G	0.35	1.12	5.43×10^{-6}	
**3q26.2**	*TERC, MYNN*	rs10936599		169,492,101			C	T	0.75	1.11	7.43×10^{-5}	2
			chr3:169539272:D	169,539,272	-	-	AT	A	0.80	1.12	6.07×10^{-6}	
**6p21.31**	*CDKN1A*	rs1321311		36,622,900			T	G	0.21	1.09	2.75×10^{-4}	3
			rs9918353	36,622,677	0.96	1.00	C	A	0.21	1.09	1.69×10^{-4}	
**8q23.3**	*EIF3H **	rs16892766		117,630,683			C	A	0.09	1.25	6.03×10^{-9}	4
			rs76316943	117,848,307	0.07	-	A	G	0.01	1.58	1.82×10^{-11}	
**8q24.21**	*MYC **	rs6983267		128,413,305			G	T	0.52	1.18	1.61×10^{-13}	5
			rs7014346	128,424,792	0.44	1.00	A	G	0.34	1.19	5.71×10^{-15}	
**10p14**	*GATA3 **	rs10795668		8,701,219			G	A	0.67	1.16	1.94×10^{-10}	4
			rs11255841	8,739,580	0.86	0.96	T	A	0.68	1.18	4.32×10^{-13}	
**10q24.2**	*SLC25A28,*	rs1035209		101,345,366			T	C	0.20	1.15	1.56×10^{-6}	6
	*NKX2-3 **		rs11190164	101,351,704	0.42	0.86	G	A	0.29	1.13	7.91×10^{-7}	
**11q13.4**	*POLD3*	rs3824999		74,345,550			C	A	0.47	1.15	8.23×10^{-11}	3
			chr11:74303133:D	74,303,133	-	-	C	CG	0.50	1.15	2.87×10^{-12}	
**11q23.1**	*FLJ45803 **	rs3802842		111,171,709			C	A	0.27	1.13	1.76×10^{-6}	7
			chr11:111172236:D	111,172,236	-	-	ATGTGCAATG	A	0.28	1.14	2.37×10^{-7}	
**12p13.32**	*CCND2 **	rs3217810	rs3217810	4,388,271	-	-	T	C	0.12	1.16	9.97×10^{-6}	1
**12q13.13**	*DIP2B, ATF1*	rs11169552		51,155,663			C	T	0.75	1.08	4.05×10^{-5}	2
			rs4768903	51,045,449	0.51	0.80	A	G	0.61	1.10	4.22×10^{-8}	
**14q22.2**	*BMP4*	rs4444235		54,410,919			C	T	0.48	1.09	7.22×10^{-5}	8
			rs35107139	54,419,106	0.64	1.00	C	A	0.40	1.10	2.41×10^{-6}	

**15q13.3**	*GREM1, SCG5*	rs4779584		32,994,756			T	C	0.19	1.19	1.45×10^{-9}	4
			rs2293582	33,010,412	0.71	0.87	A	G	0.21	1.21	2.57×10^{-11}	
**16q22.1**	*CDH1*	rs9929218		68,820,946			G	A	0.71	1.09	3.60×10^{-4}	8
			rs4783684	68,833,888	0.33	-	C	G	0.68	1.08	1.48×10^{-4}	
**18q21.2**	*SMAD7 **	rs4939827		46,453,463			T	C	0.53	1.24	7.84×10^{-21}	9
			rs7226855	46,454,048	1.00	1.00	A	G	0.55	1.25	4.09×10^{-23}	
**19q13.11**	*RHPN2,*	rs10411210		33,532,300			C	T	0.90	1.18	4.68×10^{-5}	
	*GPATCH1 **		chr19:33491901:D	33,491,901	-	-	GTAT	G	0.79	1.13	4.58×10^{-5}	
**20p12.3**	*BMP2 **	rs961253		6,404,281			A	C	0.37	1.10	4.89×10^{-5}	8
			rs57046232	6,380,344	0.80	0.93	T	A	0.37	1.11	5.05×10^{-7}	
		rs4813802		6,699,595			G	T	0.34	1.12	2.54×10^{-6}	8
			rs1015563	6,690,101	0.79	0.92	T	C	0.35	1.12	1.90×10^{-6}	
**20q13.33**	*LAMA5*	rs4925386		60,921,044			C	T	0.68	1.12	1.37×10^{-7}	2
			rs2427308	60,969,451	0.40	0.72	C	T	0.78	1.19	3.35×10^{-11}	

(B): Previously published risk loci discovered in East Asian populations. Shown for each region are the GWAS SNPs with the associated odds ratio and *P*-values for both the reported study and this study. RAF in the EUR population were obtained from the 1000 Genomes Project, and are shown in parentheses

Locus	Nearest Gene(s)	GWAS SNP	Location (bp)	Risk Allele	Alt Allele	RAF	Reported OR	Reported *P*	OR	*P*	Reference
**5q31.1**	*C5orf66*	rs647161	134,499,092	A	C	0.31 (0.66)	1.15	$2x10^{-14}$	1.06	$1.68x10^{-2}$	11
**6q25.3**	*SLC22A3*	rs7758229	160,840,252	T	G	0.25 (0.32)	1.28	$8x10^{-9}$	1.01	$6.57x10^{-1}$	12
**10q22.3**	*ZMIZ1-AS1*	rs704017	80,819,132	G	A	0.32 (0.56)	1.10	$2x10^{-8}$	1.07	$2.49x10^{-3}$	13
**10q25**	*VTI1A*	rs12241008	114,208,702	C	T	0.28 (0.10)	1.19	$2.9x10^{-8}$	1.13	$1.71x10^{-3}$	14
**11q12.2**		rs174550	61,552,680	G	A	0.59 (0.35)	1.16	$9x10^{-21}$	1.08	$1.06x10^{-3}$	13
**12p13.31**	*LOC102723767*	rs10849432	6,385,727	T	C	0.82 (0.90)	1.14	$6x10^{-10}$	1.02	$6.86x10^{-1}$	13
**12p13.33**	*WNK1*	rs12309274	975,948	T	G	0.85 (0.86)	1.11	$3x10^{-6}$	1.07	$3.64x10^{-2}$	13
**17p13.3**	*NXN*	rs12603526	800,593	C	T	0.3 (0.01)	1.10	$3x10^{-8}$	1.04	$5.71x10^{-1}$	13
**19q13.2**		rs1800469	41,860,296	G	A	0.48 (0.69)	1.09	$1x10^{-8}$	1.07	$8.68x10^{-3}$	13

REFERENCES

1. Peters et al. *Identification of Genetic Susceptibility Loci for Colorectal Tumors in a Genome-Wide Meta-analysis.* Gastroenterology, 2013. **144**(4): p. 799-807
2. Houlston et al. *Meta-analysis of three genome-wide association studies identifies susceptibility loci for colorectal cancer at 1q41, 3q26.2, 12q13.13 and 20q13.33.* Nat Gen, 2010. **42**(11): p. 973-7

3. Dunlop et al. *Common variation near CDKN1A, POLD3 and SHROOM2 influences colorectal cancer risk.* Nat Gen, 2012. **44**(7): p. 770-6
4. Tomlinson et al. *A genome-wide association study identifies colorectal cancer susceptibility loci on chromosomes 10p14 and 8q23.3.* Nat Gen, 2008. **40**(5): p. 623-30
5. Tomlinson et al. *A genome-wide association scan of tag SNPs identifies a susceptibility variant for colorectal cancer at 8q24.21.* Nat Gen, 2007. **39**(8): p. 984-8
6. Whiffin et al. *Identification of susceptibility loci for colorectal cancer in a genome-wide meta-analysis.* Hum Mol Genet, 2014. **23**(17): p. 4729-37
7. Tenesa et al. *Genome-wide association scan identifies a colorectal cancer susceptibility locus on 11q23 and replicates risk loci at 8q24 and 18q21.* Nat Gen, 2008. **40**(5): p. 631-7
8. Houlston et al. *Meta-analysis of genome-wide association data identifies four new susceptibility loci for colorectal cancer.* Nat Gen, 2008. **40**(12): p. 1426-35
9. Broderick et al*. A genome-wide association study shows that common alleles of SMAD7 influence colorectal cancer risk.* Nat Gen, 2007. **39**(11): p. 1315-7
10. Tomlinson et al. *Multiple Common Susceptibility Variants near BMP Pathway Loci GREM1, BMP4, and BMP2 Explain Part of the Missing Heritability of Colorectal Cancer.* PLoS Genetics, 2011. **7**(6)
11. Jia et al. *Genome-wide association analyses in East Asians identify new susceptibility loci for colorectal cancer.* Nat Genet, 2013. **45**(2): p191-6
12. Cui et al. *Common variant in 6q26-q27 is associated with distal colon cancer in an Asian population. Gut,* 2011. **60**(6): p799-805
13. Zhang et al. *Large-scale genetic study in East Asians identifies six new loci associated with colorectal cancer risk.* Nat Genet, 2014. **46**(6): p533-42
14. Wang et al. *Trans-ethnic genome-wide association study of colorectal cancer identifies a new susceptibility locus in VTI1A.* Nat Commun, 2014. 5: p4613

Supplementary Table 4: Relationship between SNP genotype and gene expression from RNA-seq data in 223 colon and 75 rectal cancers.

Variant	Proxy for	Gene	Probe	*P*-Colon	*P*-Rectal
rs2744753	rs72647484	*WNT4*	WNT4.54361	0.59	0.87
rs2744753	rs72647484	*CDC42*	CDC42.998	0.95	0.38
rs10904850	rs10904849	*CUBN*	CUBN.8029	0.62	0.51
rs16941835	-	*FOXL1*	FOXL1.2300	0.71	0.09

Supplementary Table 5: Summary of genomic annotation by CADD. Data are shown for rs72647484, rs16941835 and rs10904849 (in red) and proxy SNPs (r2>0.8 in 1000 Genomes phase 1 data) demonstrating evidence of histone marks, transcription factor occupancy and evolutionary conservation (GERP and PhastCons). Also indicated are RegulomeDB and CADD scores.

Chr	Position	SNP	r^2	D'	Ref	Alt	MAF	Type	PhCons[a]	GERP[b]	$H_3K_{27}Ac$	H_3K_4Me	$H_3K_4Me_3$	TFBS	Gene	CADD Score[c]	Regulome DB score[d]
**1**	**22587728**	**rs72647484**	**1.00**	**1.00**	**T**	**C**	**0.09**	**Regulatory Feature**	**0.02**	**0.84**	**4.00**	**5.32**	**2.88**		**NA**	**2.58**	**6**
1	22590009	rs72647488	0.93	1.00	G	A	0.08	Intergenic	0.00	-1.52	6.00	7.96	3.00		MIR4418	1.64	No data
1	22590125	rs72647489	0.93	1.00	**T**	**C**	0.08	Intergenic	0.17	-0.99	7.80	7.56	2.76		MIR4418	8.21	No data
10	16995872	rs7903108	0.94	0.99	G	C	0.33	Intronic	0.12	-0.43	5.12	15.32	2.84		CUBN	2.06	No data
10	16997246	rs10904848	0.96	1.00	C	T	0.33	Intronic	0.01	-1.33	2.00	4.96	2.16		CUBN	0.70	6
**10**	**16997266**	**rs10904849**	**1.00**	**1.00**	**G**	**T**	**0.32**	Intronic	**0.00**	**-0.45**	**2.88**	**5.00**	**2.00**		**CUBN**	**0.19**	**No data**
10	16997707	rs10904850	0.95	1.00	G	A	0.33	Intronic	0.01	-1.70	3.00	4.00	3.44		CUBN	3.08	No data
10	16997827	rs10904851	0.95	1.00	**G**	A	0.33	Intronic	0.00	-5.71	2.48	7.92	2.00		CUBN	0.06	5
10	16998234	rs10904852	0.95	1.00	A	G	0.33	Intronic	0.47	1.20	1.00	1.20	3.00		CUBN	11.53	No data
10	16998503	rs11254299	0.95	1.00	A	T	0.33	Intronic	0.00	-2.82	4.00	2.00	2.00		CUBN	1.71	No data
10	16999350	rs7071576	0.95	1.00	G	**A**	0.33	Intronic	0.00	0.59	2.00	6.00	2.00		CUBN	0.74	3a
10	17003751	rs28499209	0.95	1.00	C	T	0.33	Intronic	0.05	0.00	1.00	1.00	1.80		CUBN	1.69	No data
16	86691273	rs59689370	0.91	0.97	**C**	A	0.21	Transcript	0.25	0.16	2.04	3.00	1.00		RP11-58A18.1	5.74	No data
**16**	**86695720**	**rs16941835**	**1.00**	**1.00**	**G**	**C**	**0.21**	**Regulatory Feature**	**0.04**	**1.11**	**95.44**	**57.32**	**4.00**		**NA**	**0.03**	**No data**
16	86696224	rs36005190	0.99	1.00	G	C	0.21	Transcript	0.00	-8.15	50.80	40.28	4.00		RP11-58A18.1	0.08	5
16	86697008	rs7199483	0.98	0.99	C	T	0.21	Transcript	0.00	-0.10	22.20	26.72	3.00		RP11-58A18.1	3.04	5
16	86699767	rs899245	0.82	0.97	**T**	A	0.24	Transcript	0.00	-2.29	9.12	18.96	3.32		RP11-58A18.1	1.63	No data
16	86700030	rs899244	0.86	0.97	C	T	0.23	Transcript	0.00	0.14	5.52	9.72	2.68		RP11-58A18.1	1.82	No data
16	86701111	rs35295491	0.85	0.97	G	A	0.23	Transcript	0.08	0.31	7.32	17.00	8.00		RP11-58A18.1	4.92	6
16	86701426	rs7203324	0.85	0.97	C	G,T	0.23	Transcript	0.16	0.05	10.00	11.00	3.00		RP11-58A18.1	3.43,4.05	6
16	86701519	rs34740226	0.85	0.97	**A**	G	0.23	Transcript	0.00	-0.09	5.80	8.60	1.00		RP11-58A18.1	0.59	6

16	86701780	rs7184245	0.84	0.96	G	A	0.23	Regulatory Feature	0.00	0.84	18.08	22.44	5.00	2	NA	2.51	4
16	86702158	rs7186373	0.85	0.97	C	T	0.23	Regulatory Feature	0.14	1.60	36.92	28.88	15.72	1	NA	4.59	5
16	86702285	rs7192259	0.85	0.97	**T**	A	0.23	Transcript	0.00	-0.01	22.80	28.04	7.40		RP11-58A18.1	2.77	5
16	86702431	rs12443866	0.85	0.97	C	T	0.23	NonCoding Transcript	0.00	-1.64	7.44	19.64	3.52		RP11-58A18.1	0.70	No data
16	86702768	rs12928518	0.85	0.97	T	C	0.23	Intergenic	0.01	0.46	5.00	28.08	4.20		RP11-58A18.1	1.37	5
16	86703086	rs12923555	0.85	0.97	G	C	0.23	Intergenic	0.00	-1.70	5.08	17.00	5.88		RP11-58A18.1	0.15	5
16	86703949	rs62042090	0.85	0.97	C	T	0.23	Regulatory Feature	0.01	0.95	7.28	13.76	9.56	1	NA	6.29	5
16	86705372	rs11117200	0.85	0.97	G	A	0.23	Intergenic	0.00	-3.85	8.12	23.92	5.00		RP11-58A18.1	0.84	4
16	86706795	rs35285681	0.85	0.97	G	T	0.23	Intergenic	0.30	1.67	11.44	21.40	3.00	2	RP11-58A18.1	8.49	4
16	86706867	rs60933831	0.85	0.97	T	A	0.23	Intergenic	0.14	-1.28	6.40	16.40	2.08	2	RP11-58A18.1	4.03	2b
16	86708914	rs16941856	0.85	0.96	C	T	0.23	Intergenic	0.22	0.14	3.00	9.56	2.00		NA	5.63	5
16	86709958	rs58938680	0.85	0.96	GT	G	0.23	Regulatory Feature	0.14	1.35	25.32	36.40	2.56	3	NA	5.19	2b

Chr,chromosome; Ref, reference allele; Alt, alternate allele; DNase, DNase hypersensitivity; GERP, Genomic Evolutionary Rate Profiling; $H_3K_{27}Ac$, mark found near active regulatory elements; H_3K_4Me, mark found near regulatory elements; $H_3K_4Me_3$, mark found near promoters; TFBS, number of transcription factor binding sites

[a]PhastCons scores >0.3 indicative of conservation

[b]GERP scores indicative >2 are indicative of evolutionary constraint

[c]CADD score >10 indicate a variant is likely deleterious

[d]RegulomeDB scores: 2b, TF binding + any motif + DNase Footprint + DNase peak; 3a, TF binding + any motif + DNase peak; 4, TF binding + DNase peak; 5, TF binding or DNase peak; 6, other.

Supplementary Table 6: Pathways overrepresented in GWAS regions. All genes within the LD block containing each tagSNP, or linked to the SNP through functional experiments, were uploaded to the NCI pathway interaction database. Displayed are pathways containing three or more genes.

Pathway Name	Biomolecules in Group 1	P-value
Alpha6 beta4 integrin-ligand interactions	LAMA5, LAMB2, LAMC1, LAMC2	1.88E-07
a6b1 and a6b4 Integrin signaling	CDH1, LAMA5, LAMB2, LAMC1, LAMC2	3.00E-06
BMP receptor signaling	BMP2, BMP4, GREM1, SMAD7	6.10E-05
Beta1 integrin cell surface interactions	LAMA5, LAMB2, LAMC1, LAMC2	4.01E-04
Regulation of nuclear SMAD2/3 signaling	CDKN1A, LAMC1, MYC, SMAD7	7.25E-04
Stabilization and expansion of the E-cadherin adherens junction	AQP5, CDH1, LIMA1	1.39E-03
Regulation of nuclear beta catenin signaling and target gene transcription	CCND2, CDH1, MYC	7.52E-03

Supplementary Table 7: Relationship between SNP genotype and sex, age at diagnosis of CRC, tumor site (rectal [ICD9:154], colonic [ICD9:153]), stage, family history of CRC (≥1 affected first degree relative), MSI status and both KRAS and BRAF mutant status.

			***P*-values**		
**Feature**	**No Samples**	**Studies**	**rs72647484**	**rs10904849**	**rs16941835**
Age	4,325	UK1, Scotland1, VQ58 and COIN	0.77	0.51	0.97
Gender	6,622	All	0.41	0.15	0.16
Site	2,868	VQ58 and COIN	0.82	0.60	0.39
Stage	1,401	UK1 and VQ58	0.76	0.42	0.23
MSI	1,260	UK1 and COIN	0.14	0.96	0.92
KRAS	1,623	COIN	0.03	0.86	0.08
BRAF	1,470	COIN	0.47	0.11	0.63

Supplementary Figure 1:

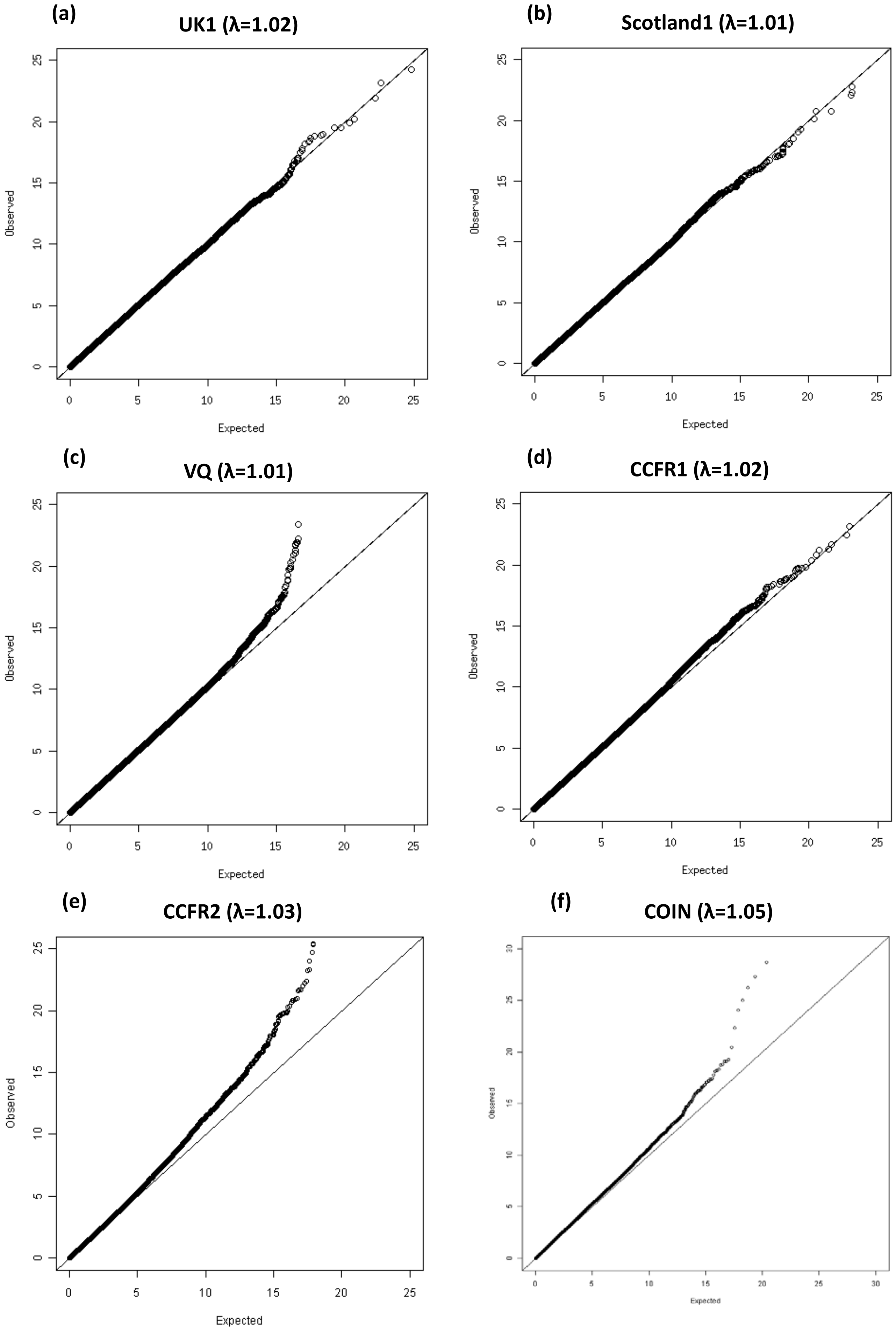

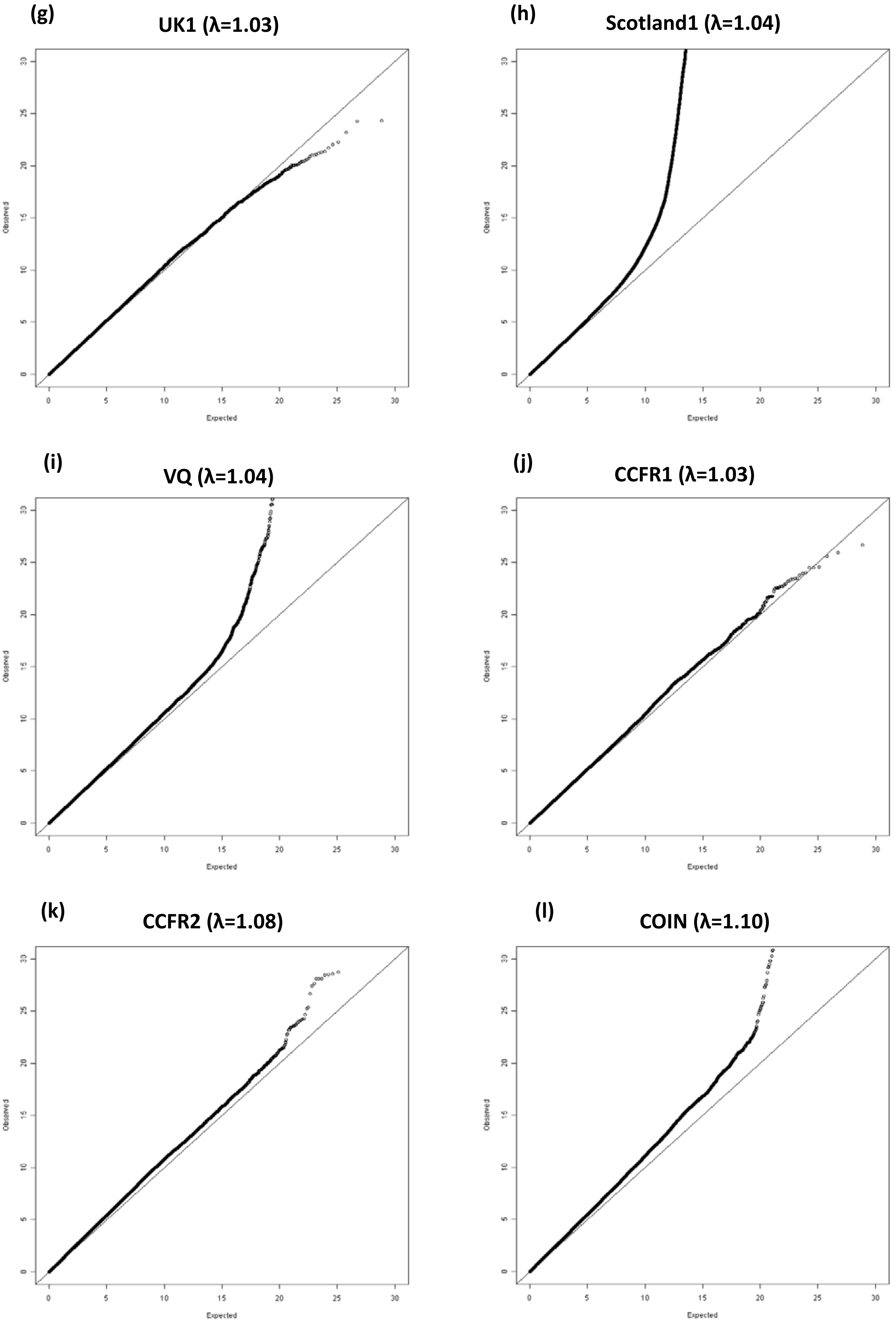
(g)
UK1 (λ=1.03)
(h)
Scotland1 (λ=1.04)
(i)
VQ (λ=1.04)
(j)
CCFR1 (λ=1.03)
(k)
CCFR2 (λ=1.08)
(l)
COIN (λ=1.10)
Observed
Expected
0
5
10
15
20
25
30

(m)

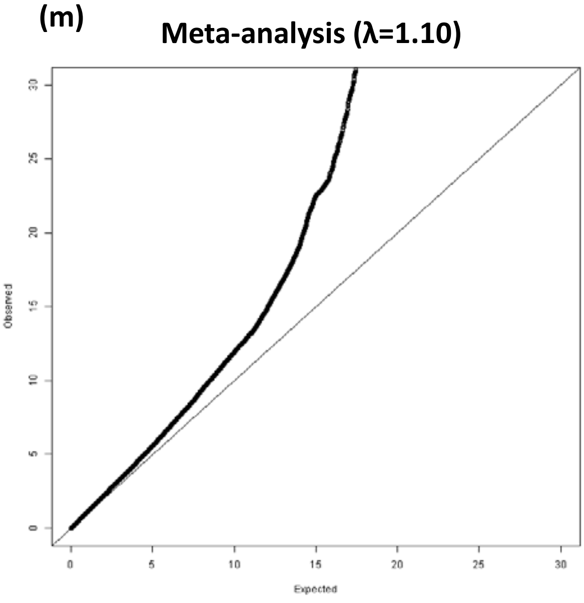

Supplementary Figure 2:

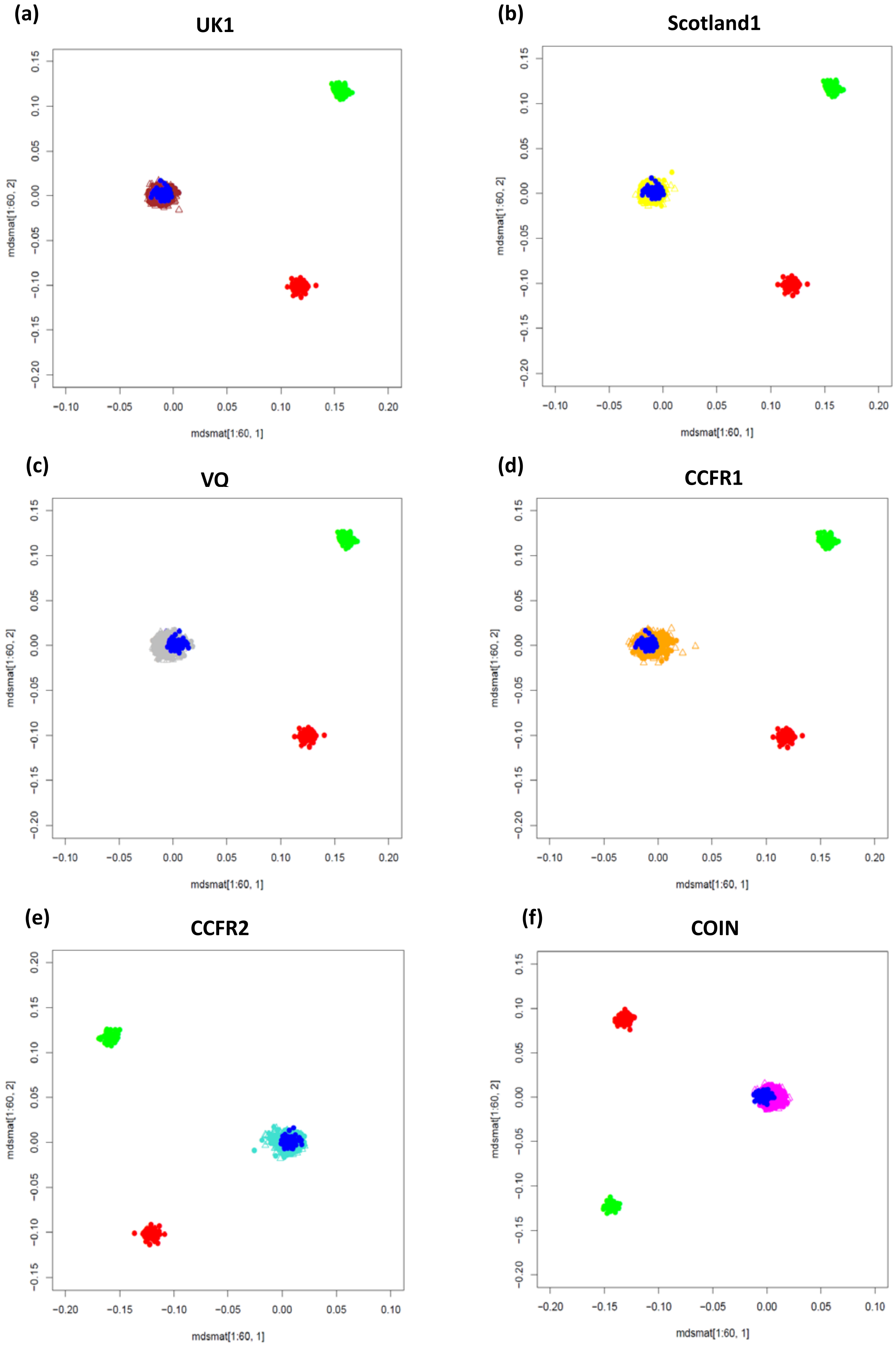

	UK1	Scotland1	VQ58	CCFR1	CCFR2	COIN
**pre-QC**	**940 cases 965 controls**	**1,012 cases 1,012 controls**	**1,800 cases 2,690 controls**	**1,290 cases 1,055 controls**	**796 cases 2,236 controls**	**2,244 cases 2,162 controls**
Call rate	15	15	0	84	0	122
Ethnicity	54	9	0	67	2	130
Relatedness	26	9	9	13	0	4
Sex discrepancy	3	15	1	4	1	8
Other	17	5	0	3	0	30
**post-QC**	**890 cases 900 controls**	**973 cases 998 controls**	**1,794 cases 2,686 controls**	**1,175 cases 999 controls**	**795 cases 2,234 controls**	**1,950 cases 2,162 controls**

Supplementary Figure 3: Details of the quality control filters applied to each GWAS. Samples were excluded due to call rate (<95% or failed genotyping), Ethnicity (principle components analysis or other samples reported to be not of white, European descent), Relatedness (any individuals found to be duplicated or related within or between data sets through IBS), sex discrepancies or others (cases found to contain a previously reported susceptibility allele, controls with a 1st degree relative with CRC, low concordance of genotyping in duplicates or samples which have been subsequently withdrawn from a study).
